# Iron-induced oligomerization of human FXN^81-210^ and bacterial CyaY frataxin and the effect of iron chelators

**DOI:** 10.1371/journal.pone.0188937

**Published:** 2017-12-04

**Authors:** Eva-Christina Ahlgren, Mostafa Fekry, Mathias Wiemann, Christopher A. Söderberg, Katja Bernfur, Olex Gakh, Morten Rasmussen, Peter Højrup, Cecilia Emanuelsson, Grazia Isaya, Salam Al-Karadaghi

**Affiliations:** 1 Center for Molecular Protein Science, Department of Biochemistry and Structural Biology, Lund University, Lund, Sweden; 2 Biophysics Department, Faculty of Science, Cairo University, Giza, Egypt; 3 Departments of Pediatric and Adolescent Medicine and Biochemistry and Molecular Biology, Mayo Clinic, College of Medicine, Rochester, Minnesota, United States of America; 4 Department of Biochemistry and Molecular Biology, University of Southern Denmark, Odense, Denmark; Sant Joan de Déu Children's Hospital, SPAIN

## Abstract

Patients suffering from the progressive neurodegenerative disease Friedreich’s ataxia have reduced expression levels of the protein frataxin. Three major isoforms of human frataxin have been identified, FXN^42-210^, FXN^56-210^ and FXN^81-210^, of which FXN^81-210^ is considered to be the mature form. Both long forms, FXN^42-210^ and FXN^56-210^, have been shown to spontaneously form oligomeric particles stabilized by the extended N-terminal sequence. The short variant FXN^81-210^, on other hand, has only been observed in the monomeric state. However, a highly homologous *E*. *coli* frataxin CyaY, which also lacks an N-terminal extension, has been shown to oligomerize in the presence of iron. To explore the mechanisms of stabilization of short variant frataxin oligomers we compare here the effect of iron on the oligomerization of CyaY and FXN^81-210^. Using dynamic light scattering, small-angle X-ray scattering, electron microscopy (EM) and cross linking mass spectrometry (MS), we show that at aerobic conditions in the presence of iron both FXN^81-210^ and CyaY form oligomers. However, while CyaY oligomers are stable over time, FXN^81-210^ oligomers are unstable and dissociate into monomers after about 24 h. EM and MS studies suggest that within the oligomers FXN^81-210^ and CyaY monomers are packed in a head-to-tail fashion in ring-shaped structures with potential iron-binding sites located at the interface between monomers. The higher stability of CyaY oligomers can be explained by a higher number of acidic residues at the interface between monomers, which may result in a more stable iron binding. We also show that CyaY oligomers may be dissociated by ferric iron chelators deferiprone and DFO, as well as by the ferrous iron chelator BIPY. Surprisingly, deferiprone and DFO stimulate FXN^81-210^ oligomerization, while BIPY does not show any effect on oligomerization in this case. The results suggest that FXN^81-210^ oligomerization is primarily driven by ferric iron, while both ferric and ferrous iron participate in CyaY oligomer stabilization. Analysis of the amino acid sequences of bacterial and eukaryotic frataxins suggests that variations in the position of the acidic residues in helix 1, β-strand 1 and the loop between them may control the mode of frataxin oligomerization.

## Introduction

Frataxin (FXN) is a 210 amino acid nucleus-encoded mitochondrial protein highly conserved in eukaryotic organisms and in bacteria. In humans, abnormally large GAA repeats in the first intron of the gene coding for FXN result in low levels of frataxin expression, and have been linked to the autosomal recessive hereditary neurodegenerative disease Friedreich’s ataxia (FRDA). FRDA is characterized by progressive gait and limb ataxia, hypertrophic cardiomyopathy and diabetes [1, 2]. At the cellular level frataxin deficiency has been linked to up-regulation of iron uptake, high levels of reactive oxygen species, and decreased levels of iron-sulfur cluster (ISC) and heme synthesis [2, 3].

Three major isoforms of human frataxin have been identified and include FXN^42-210^, FXN^56-210^ and FXN^81-210^, of which FXN^81-210^ is the most abundant form in mitochondria and is considered to be the mature form of the protein [4–8]. All above frataxin isoforms have been observed in mitochondrial extracts from human heart cells [9], human lymphoblastoid cells [8], as well as upon expression of frataxin in *E*. *coli* and in yeast cells [5]. The isoforms are created by the cleavage of the precursor FXN^1-210^ by the mitochondrial processing peptidase after the import of the protein into the mitochondrial matrix. One of the distinctive features of FXN^42-210^ and FXN^56-210^ is their ability to spontaneously assemble into oligomeric, nano-caged structures [5, 8, 10]. In a way similar to that of ferritin, oligomeric FXN^56-210^ has been shown to use the ferroxidation reaction to build a ferrihydrite mineral core inside the particles [5, 11]. On the other hand, oligomeric FXN^42-210^ has been shown to form stable contacts with components of the ISC assembly machinery NFSD1•ISD11 and ISCU, and to donate both Fe^2+^ and Fe^3+^ to ISC assembly [8, 12]. In addition, the metallochaperone function has been shown to serve in donating Fe^2+^ to heme synthesis and aconitase repair [12–16]. In contrast to FXN^42-210^ and FXN^56-210^, FXN^81-210^ has been suggested to be mostly monomeric, and has been found to interact with the assembled ISC synthesis machinery and donate Fe^2+^ to ISC assembly in an iron-dependent process [8, 17, 18]. It has been suggested that the metallochaperone function of frataxin depends on the ability of both monomeric and assembled protein to bind ferrous iron and maintain it in an available form for up to 60 min, which is much longer than the normal auto-oxidation time of iron in solution [10].

Even yeast Yfh1 and bacterial CyaY frataxin have been shown to have metallochaperone function and to interact with components of the ISC and heme synthesis machinery [19–25]. Both proteins also form oligomeric species, although in contrast to FXN^42-210^ and FXN^56-210^, they require iron to induce oligomerization [5, 19, 26–30].

A number of studies have addressed the issue of iron binding by frataxin [5, 10, 13, 28, 31–33]. Using isothermal titration calorimetry (ITC), it was shown that yeast and bacterial frataxin CyaY under anaerobic conditions bind two ferrous ions/monomer [25, 28, 32]. For human frataxin FXN^81-210^, using ITC and tryptophan fluorescence measurements, the iron binding capacity was determined to be 6–7 atoms/monomer at aerobic conditions (ferric and ferrous iron were measured separately) [13]. However, it should be noted that the authors of this study used a His-tagged protein, which may have affected the measured metal binding capacity. A more recent study showed that the number of bound metal atoms (ferrous, ferric and cobalt were tested) was 3/monomer, with His86 being involved in the highest affinity metal binding site [33]. The longer FXN^56-210^ isomer, which is spontaneously assembled into oligomeric particles upon expression in *E*. *coli*, was shown to bind 7–10 iron atoms/molecule [5]. Additional insights into iron binding by yeast and human frataxin were obtained using X-ray absorption spectroscopy (XAS) [16, 32]. The experiments showed that iron was bound in a symmetric six-coordinated nearest-neighbor oxygen/nitrogen ligand environment. This was in agreement with NMR spectroscopic data, which showed that histidine, aspartate and glutamate residues from the acidic helix α1 and strand β2 of the molecule were involved in metal binding [31, 33, 34]. In addition, Nichol et al. [11], also by using XAS, showed that frataxin iron core is similar in structure to ferritin iron core, and contains small ferrihydrite crystallites composed of ferric oxide/hydroxide octahedra.

Common to all these studies is that they show that frataxin can bind both ferrous and ferric iron. It has also been shown that iron binding affinity is rather weak with the dissociation constants (*K*_*D*_) for Fe^2+^ binding to CyaY of about 4 μM, and for human frataxin about 11.7 μM and 55.0 μM for Fe^3+^ and Fe^2+^, respectively [13, 28]. As noted earlier, this relatively low affinity is common for metallochaperones, which need to give away the iron upon binding to their target [25, 28].

The oxidation state of iron in the presence of frataxin has also been assessed in a number of studies. In the case of CyaY, the rate of iron oxidation was measured in the absence and presence of the protein using iron-to-protein ratio of 6:1 [28]. The experiments showed that at aerobic conditions the rate of iron oxidation in the absence of protein was twice as fast as the rate in the presence of CyaY, indicating that CyaY binds iron and maintains its oxidation state. In the presence of H_2_O_2_ as oxidant, the oxidation occurred rapidly and completely, with high attenuation of iron-catalyzed hydroxyl radical generation [28]. Similarly, it has been shown that both assembled and monomeric frataxin FXN^56-210^ as well as its degradation product FXN^78-210^ could keep iron available to the ferrous iron chelators 2,2’-bipyridine (BIPY) and to the ferrochelatase reaction [10]. After up to 60 min, about 3–5% of the iron was still available for chelation. Also in this case, in the absence of frataxin iron in the buffer was rapidly oxidized and precipitated after about 10 min.

We have earlier used electron microscopy (EM), small-angle X-ray scattering (SAXS) and X-ray crystallography in the study of frataxin oligomers. The studies showed that the stabilization of oligomeric structures of yeast and human frataxin depends to a large extent on the long, normally disordered N-terminal region of the molecules [12, 29, 30, 35, 36]. On the other hand, oligomers of *E*. *coli* CyaY, a protein that lacks the long N-terminal extension, form only in the presence of iron at anaerobic as well as aerobic conditions [28]. These observations raise the question on the nature of the interactions that control and stabilize oligomerization of the shorter forms of frataxin and the role of iron in stabilizing these interactions.

Here, using dynamic light scattering (DLS), SAXS, EM and cross linking mass spectrometry (MS), we compare the effect of iron on the oligomerization state of the two short forms of frataxin—human frataxin FXN^81-210^ and *E*. *coli* CyaY. We show that at aerobic conditions at iron-to-protein ratios above 6:1 small ring-shaped structures of FXN^82-210^ are formed in solution. Based on EM and cross-linking data, we propose a model for stabilization of these oligomers by iron. We also show that ferric iron chelators deferoxamine mesylate (DFO) and 3-Hydroxy-1,2-dimethyl-4(1*H*)-pyridone (deferiprone), suggested earlier for use in the treatment of FRDA [37], are able to dissociate CyaY oligomers, but promote FXN^81-210^ oligomerization.

## Materials and methods

### Protein expression and purification

Human frataxin isoform 81–210 (FXN^81-210^) was recombinantly expressed in *E*. *coli* and purified as previously described [8], with a few modifications. Complete EDTA-free inhibitor mix (Roche) was added to the bacterial cells, which were subsequently disrupted using a French press. CyaY was expressed and purified according to Bedekovics et al. [38], except that the second-step anion exchange using Macro-Prep High Q was not performed. During the following experiments, the buffer solution consisted of 10 mM HEPES, pH 7.3, 150 mM NaCl, 5 mM Mannitol (HN150) for FXN ^81–210^ and 20 mM HEPES, pH 7.3, 100 mM NaCl (HN100) for CyaY.

### Dynamic light scattering

The DLS method, also referred to as photon correlation spectroscopy, is a technique used for measuring particle size in the sub-micron range. The method is based on measuring the Brownian motion, which defines the translational diffusion coefficient (D) of particles in solution. D depends on the size of the particles, defined as the hydrodynamic radius, which is essentially the radius of a sphere that has the same D value as the particles studied. However, it should always be kept in mind that the hydrodynamic radius is only an approximation, since proteins and protein oligomers rarely have the form of a perfect sphere. The Brownian motion is measured by determining the rate at which the intensity of scattered light fluctuates. Practically, the measurements are performed using a device called autocorrelator, which measures the correlation function defining the degree of similarity between two signals, or one signal with itself, at varying time intervals. Since after some time the signal will change, the correlation to the initial signal at time zero will be reduced until there will be no correlation. This correlation decay time, in turn, is proportional to the diffusion speed, which of course depends on particle size. By fitting a single exponential function to the correlation function the mean size and width of the distribution (polydispersity) can be obtained. By fitting multiple exponents the distribution of particle sizes can also be obtained. As noted in the manual for the instrument used in this study, the first-order result from a DLS experiment is an intensity distribution of particles sizes, although this is converted to volume distribution, using Mie theory (for details see https://www.malvern.com/). The volume distribution, on which the analysis made in this work is based, describes the relative percentage of multiple components in the sample based on their mass or volume. It should be kept in mind that as a result of a number of assumptions, which have to be made during the calculations, the obtained distribution is not an absolute measure of the oligomerization state of the system and should be used primarily for comparative purposes.

In our experiments we used the Zetasizer Nano S instrument (Malvern Instruments Ltd, UK) for the DLS measurements. The experiments were run at a 173° scattering angle and a laser working at 633 nm. A ZEN2112 quartz cuvette was used with a 30 μl reaction mixture for each measurement. For the study of aerobic metal-induced oligomerization, FXN^81-210^ at a concentration of 2.5 mg/ml (0.17 mM) and CyaY at 2 mg/ml (0.16 mM) were mixed with ammonium iron (II) sulfate, (NH_4_)_2_Fe(SO_4_)_2_·6H_2_O. For FXN^81-210^ the ammonium iron (II) sulfate concentration range applied was 0.35–1.75 mM. The Fe^2+^: protein molar ratios used in the following experiments were 2:1, 4:1, 5:1, 6:1, and 10:1 for FXN^81-210^. As soon as the (NH_4_)_2_Fe(SO_4_)_2_·6H_2_O was added to FXN^81-210^, the sample was mixed and quickly spun down for 2 min before transfer to the DLS cuvette and start of the measurements. First measurement was recorded after approximately 5 min of incubation. DLS profiles were recorded every 5 min for 2 h. After each measurement, the solution samples were stirred to prevent sedimentation. Solutions for measuring Fe^3+^—dependent oligomerization were prepared as described by Yoon & Cowan [13]: Ferric ion solutions were prepared by adding 0.5 M FeCl3 in 0.05 M HCl into 100 mM pH 7.5 HEPES buffer to obtain a desirable range of ferric ion concentration. To avoid solubility problems for ferric ion, a concentrated stock was prepared in acidic solution.

For CyaY, the iron concentration range was 0.16 mM–1.64 mM, and the Fe^2+^: protein molar ratios used in the experiments were 1:1, 2:1, 4:1, 6:1, 8:1, and 10:1. DLS profiles were recorded after 1 h of incubation. All FXN^81-210^ incubations were performed at room temperature, and at 30°C for CyaY, as previously described [30]. Solutions were centrifuged at 17500 *g* for 10 min prior to DLS measurements.

For experiments with chelators, we used the Fe^3+^-specific chelators DFO (Sigma-Aldrich) and deferiprone (Sigma-Aldrich), also used in clinical trials for possible treatment of FRDA patients [39]. In addition, the Fe^2+^-specific chelator BIPY was used. The proteins were incubated for 15 min with ammonium iron (II) sulfate before the addition of the chelators at 3 times the amount of iron (molar ratio). All solutions were centrifuged at 17500 *g* for 10 min prior to DLS measurements. Freshly prepared chelator solutions were used in all experiments. DLS profiles were recorded as described above. For the samples with deferiprone, measurements were also made after 24 h of the initial incubation with iron.

### Size exclusion chromatography (SEC) of iron-loaded FXN^81-210^

FXN^81-210^ at a concentration of 10 mg/ml (0.70 mM) was mixed with buffer containing ammonium iron (II) sulfate to a final Fe^2+^: protein molar ratio of 10:1 (7.0 mM) and incubated at room temperature for 30 min and 1 h. The samples were subsequently centrifuged at 14000 rpm for 10 min before being applied to a 10/300 GL Superdex 200 gel filtration column (GE Healthcare) pre-equilibrated with HN150 buffer and operated at a flow rate of 0.5 ml/min at room temperature. Protein elution was followed at 280 nm.

To study the effect of the chelators on the oligomers, 4 mg/ml (0.28 mM) of FXN^81-210^ was incubated at room temperature with ammonium iron (II) sulfate at the Fe^2+^: protein molar ratio of 6:1 (1.68 mM) for 15 min. After this each chelator was added at 3 times the amount of iron and further incubated for 15 min before applying to the Superdex 200 gel filtration column operated at the flow rate of 0.5 ml/min at room temperature. In the case of the experiments with DFO, protein concentration of 7 mg/ml (0.49 mM) was used.

### Protein preparation for EM

Samples of FXN^81-210^ were applied directly to a 400-mesh carbon-coated copper grid (EMS) pre-incubated for 1 min in HN150. After 1 min, excess protein sample was blotted and the grid was washed for 3 s with sterile water. Excess water was removed by blotting. For staining, 1% (w/v) uranyl acetate was applied to the grid, and after 30 s excess stain was blotted. The grid was left to dry for approximately 30 min before it was inserted into the sample holder of a Philips CM120 transmission electron microscope equipped with a GATAN GIF 100 energy filter and a GATAN 791 CCD camera (1024x1024 pixels). All images were taken at 55000x magnification. For more uniform spread of the protein sample on the grids, glow discharge was applied and in addition 0.01% Triton-X-100 was added to the dilution mix.

### Image processing and reference free 2D class averaging

Image processing was performed using the EMAN2 software package [40]. Reference-free 2D class averages were generated using 1265 ring-shaped particles, with each class average containing 20–90 particles. The program Digital Micrograph^™^ (DM) by Gatan Inc (ref: http://www.gatan.com/products/tem-analysis/gatan-microscopy-suite-software) was used to determine the dimensions of the particles on the transmission electron microscopy (TEM) micrographs.

### SAXS measurements and modeling

For the SAXS experiments with FXN^81-210^, protein concentrations of 20, 10, and 7.5 mg/ml were used. The samples were centrifuged for 10 min at 10000 rpm prior to measurements. No radiation damage was detected in the samples.

SAXS data were collected at the EMBL X33 beamline on the storage ring DORIS III (DESY). Data were normalized to the intensity of the transmitted beam, and scattering of the buffer was subtracted. The processing was done using the ATSAS software package [41, 42]. Forward scattering *I*(0) and the radius of gyration *R*_g_ were evaluated using the Guinier approximation. These parameters, as well as the maximum particle dimension *D*_max_, were also computed from the entire scattering patterns using the program GNOM [43], which calculates the distance distribution function, *P*(r). Molecular weight estimates were made using bovine serum albumin (BSA) as a standard or from the excluded volume of the hydrated particle (the Porod volume *V*_p_), computed as reported by Porod [44]. A total **of 20 *ab initio* models were** calculated using the program GASBOR [45], and averaged using the DAMAVER suite of programs [46]. The nine N-terminal residues of FXN^81-210^ missing in the crystal structure (PDB ID 1EKG, [47]) were modelled using the program CORAL [42].

### Crosslinking of iron-induced oligomers

For the investigation of subunit arrangement in the metal-induced oligomers, FXN^81-210^ at a concentration of 4 mg/ml (0.27 mM) was mixed with ammonium iron (II) sulfate at Fe^2+^: protein molar ratio of 6:1 and incubated for 5 min at room temperature before H_2_O_2_ at a final concentration of 10 mM was added. Incubation continued for 45 min before adding the lysine-specific crosslinker, BS^2^G (bis(sulfosuccinimidyl)-glutarate). The crosslinker was dissolved in distilled water to a concentration of 30 mM just before use and added to the protein solution at 40:1 molar ratio, after which the reaction was incubated at room temperature for 15 min. The crosslinking reaction was quenched by adding ammonium bicarbonate (final concentration of 24 mM). The crosslinked protein sample was separated by SDS-PAGE and the bands corresponding to crosslinked di-, tri-, and tetrameric FXN^81-210^ were excised and subjected to in-gel digestion with trypsin before analysis.

A similar approach was used for bacterial frataxin CyaY—at a concentration of 8 mg/ml (0.65 mM) the protein solution was mixed with ammonium iron (II) sulfate at iron: protein molar ratio of 6:1 and subsequently incubated for 20 min at room temperature before adding the crosslinker. BS^2^G was added to the protein in 5:1 molar ratio and incubated at room temperature for 15 min. The sample was further processed as described for FXN^81-210^, and bands corresponding to crosslinked dimeric CyaY were analyzed.

Gel slices (1x1 mm) were washed twice in 50 mM ammonium bicarbonate/50% ethanol to remove the Coomassie blue stain and dehydrated in 100% ethanol, followed by reduction and alkylation (10 mM DTT at 37°C for 30 min, 55 mM iodoacetamide at room temperature, and darkness for 30 min). This was followed by repeated washing and dehydration. Digestion was performed by adding 50 mM ammonium bicarbonate with 12 ng/μl sequencing-grade modified trypsin (Promega, Madison, WI, USA), followed by incubation on ice for 1 h. Thereafter, 50 mM ammonium bicarbonate was added to cover the gel pieces and prevent dehydration during overnight digestion at 37°C. On the next day, the peptide-containing solution above the gel slices was withdrawn, and peptides were extracted from the gel slices by adding a 2:1 mixture of 100% acetonitrile and 1% trifluoroacetic acid (TFA) for 2 h. Extracted peptides were pooled with the overnight digestion solution.

### Mass spectrometry

Peptides were subjected to reversed phase nano-LC coupled to an LTQ-Orbitrap Velos Pro mass spectrometer (Thermo Fisher Scientific, Stockholm, Sweden) equipped with a nanoEasy spray ion source (Proxeon Biosystems, Odense, Denmark). The chromatographic separation was performed at 40°C on a 15 cm (75 μm i.d.) EASY-Spray column packed with 3 μm resin (Thermo Fisher Scientific, Stockholm, Sweden). The nanoHPLC intelligent flow control gradient was created by solvent A (0.1% (v/v) FA in water) and solvent B (0.1% (v/v) FA in 100% (v/v) acetonitrile), as follows: 5%–20% for 60 min, 20%–40% for 30 min and 40–90% for 15 min, and constant at 90% for 5 min. A flow rate of 300 nl/min was used through the whole gradient. An MS scan (400–1400 m/z) was recorded using the Orbitrap mass analyzer set at a resolution of 60,000 at 400 m/z.

The MS was followed by data-dependent collision-induced dissociation MS/MS scans on the 10 most intense multiply charged ions, excluding charge 2^+^, in the LTQ at a 15,000 signal threshold. MSMS-spectra for 15 crosslinks are shown in the supplementary information.

### Data analysis and detection of crosslinks

Raw files typically containing 20,000 scans each were converted to MGF format by Mascot Distiller (version 2.5), and identification of crosslinked peptides was carried out using the *MassAI* software (version August 2015; http://www.massai.dk). Filtering of the primary MGF files was performed with the “MGF filter” feature to retain only the 125 most intense peaks. The “Combine scans” feature was used to improve MS/MS spectra by combining data from several scans. The following search settings were used: Fragmentation Mode CID, trypsin as a protease; two allowed missed cleavages; 10 ppm MS accuracy for peptides and 0.05 Da accuracy for crosslinked peptides; and 0.05 Da MS/MS accuracy. Additional parameters included fixed modifications C: carbamidomethylation (IAA, IAM); variable modifications: K (BS2G-H20 (x-linker)); crosslinker: BS2G (d0). Searches were performed with the filtering option “Crosslink peptide only if peptide is observed as deadend”, which reduces the number of false positives, and with the setting “Also xlink modified peptides”. The MSMS spectra of proposed crosslinks were then manually validated and accepted only if three criteria were fulfilled: intensity approximately at or above 5000, fragment ions from both peptides, and no major peaks unexplained. MS/MS spectra are included in the supplementary information. The amino acid sequences used in data analysis were for human frataxin 81–210 and *E*. *coli* CyaY (UniProt accession numbers Q16595 and P27838, respectively).

## Results

### Structural characteristics of FXN^81-210^ in solution studied by SAXS

**Fig 1:**
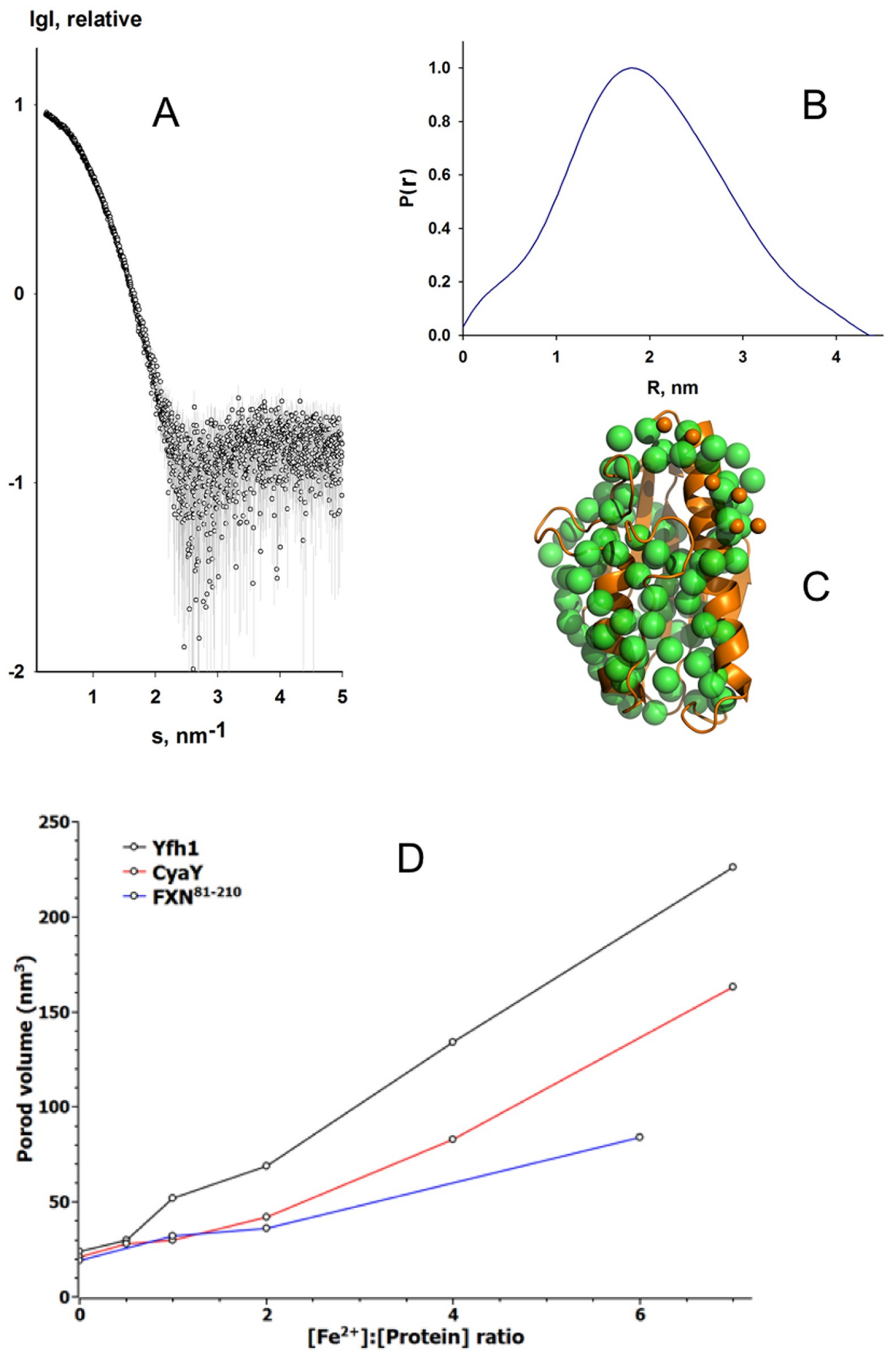
SAXS measurement of human frataxin FXN^81-210^. **A)** Small-angle X-ray scattering profile of FXN^81-210^. The experimental data are shown as circles. **B**) Pair-distribution function calculated using GNOM and the SAXS profile. **C**) Green spheres represent an *ab initio* model calculated using GASBOR, and superimposed is the X-ray crystallographic model of human frataxin (orange cartoon) residues 90–210 (PDB ID: 1EKG). The nine missing residues (orange spheres) were modeled using CORAL [42]. **D**) Porod volume for Yfh1 (black), CyaY (red), and FXN^81-210^ (blue) plotted against iron-to-protein ratio.

Prior to the study of metal-dependent oligomerization of FXN^81-210^, we used SAXS to assess the conformation and the oligomeric state of the protein in solution in the absence of added metal. No concentration effects were observed when different protein concentrations were tested prior to the SAXS measurements, thus the highest concentration was chosen for analysis (Fig 1A). The radius of gyration (*R*_g_), estimated using the Guinier approximation, was 1.4 nm. The maximum dimension, *D*_max_, was 4.5 nm, which is similar to earlier data for *E*. *coli* CyaY [30]. In the modeling and fitting of the SAXS data we used the distance distribution function P(r) (Fig 1B) and the crystal structure of human frataxin (PDB ID: 1EKG), which includes residues 90–210 [47]. The missing residues 81–89 were modeled using CORAL [42]. The calculated 10 models showed very good agreement with the experimental SAXS data, with χ^2^ between 1.06 and 1.09. We also used GASBOR [45] to calculate 20 *ab initio* models of the protein based on the SAXS data. The resulting normalized spatial discrepancy (NSD) of the *ab initio* models was in the range 0.771–0.824, which illustrated that a unique solution was identified. A comparison of the average GASBOR model with the CORAL models showed good agreement (Fig 1C). Both the CORAL and GASBOR models suggested that the flexible N-terminus of FXN^81-210^ is close in space to the core of the molecule and to the acidic helix 1 (Fig 1C).

Next, we assessed the oligomerization state of FXN^81-210^ in the presence of iron. Earlier studies on the truncated 91–210 variant and on FXN^81-210^ used gel filtration for detecting oligomers in the presence of iron [33, 48], but were unable to show oligomerization. As Fig 1D shows, for the experimental conditions used, with increasing iron content the Porod volume of the samples gradually increased. Notably, a comparison of iron-dependent volume growth for CyaY and FXN^81-210^ (Fig 1D) shows that they are very similar up to 2:1 iron-to-protein ratio. At higher ratios FXN^81-210^ oligomers have smaller volume than CyaY (and yeast frataxin) oligomers, suggesting that at similar iron concentrations CyaY may build oligomers larger than those of FXN^81-210^. Unfortunately, due to the absence of a high resolution structure of any oligomeric FXN^81-210^, at this stage we are unable to model the SAXS data.

### Iron-induced oligomerization of FXN^81-210^ and the effect of chelators studied with DLS

To gain further insights into the details of the iron-induced oligomerization of FXN^81-210^, we used DLS. The experiments were run over a period of 2 h and the effect of increasing the iron-to-protein ratio (2:1, 4:1, 5:1, 6:1, and 10:1) was studied. Similar iron-to-protein ratios have been used in earlier studies of iron binding to frataxin [5, 13, 28].

**Fig 2:**
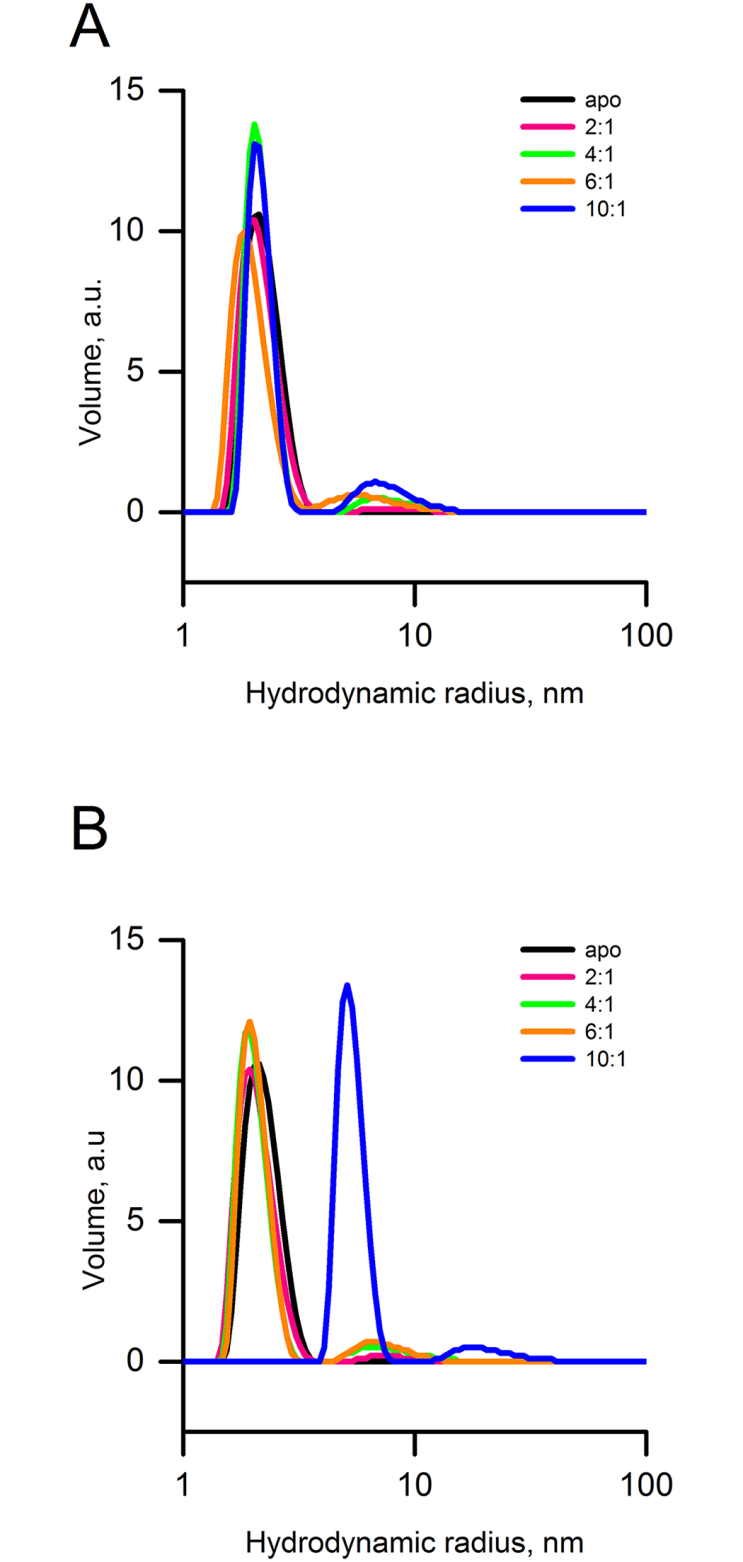
DLS studies of iron-dependent oligomerization of human frataxin FXN^81-210^. **A**) Measurements after 30 min of incubation with iron at 2:1 equivalents of iron-to-protein (magenta), 4:1 (green), and 10:1 (blue). **B**) Measurements after 60 min of incubation showing buildup of oligomers. In black is monomeric human FXN^81-210^ without the addition of iron. On the x-axis is the hydrodynamic radius of the particles and on the y-axis is the volume percentage of particles.

As in the SAXS experiments, DLS measurements showed that with no iron added, the protein was monomeric during the entire observation time (Fig 2A and 2B). As Fig 2A shows, after 30 min of incubation with iron only a small proportion of the samples was oligomerized. After 60 min of incubation, when most of the iron is expected to be oxidized [10, 28], the degree of oligomerization depended on the iron-to-protein ration. Thus, at 6:1 iron-to-protein ratio oligomers with a hydrodynamic radius in the range of 6–8.5 nm were formed, even though monomeric FXN^81-210^ was still the predominant form (Table 1). Importantly, while the monomeric peak was monodisperse, the oligomeric peak in the presence of iron showed signs of polydispersity, suggesting that a mixture of different oligomeric states was present in the solution. Although the total percentage of formed oligomers did increase with increasing relative iron concentration (from 2:1 to 6:1), it never exceeded 10% of the total protein (Fig 2B). This was in clear contrast to CyaY, for which at 6:1 iron-to-protein ratio essentially no monomers were detectable in solution (for FXN^81-210^ loss of monomers was observed at 10:1 iron-to-protein ration, see below).

**Table 1:** Average size and distribution of particles of FXN^81-210^ obtained from DLS measurements and the effect of iron chelators.

Hydrodynamic radius (nm)	1.6–2.1	6–8.5	8.5–14	15–25	< 100
FXN^81-210^ Apo (%)	100	-	-	-	-
6:1 (Fe^2+^: FXN^81-210^, %)	92	8	-	-	-
6:1 + deferiprone (%)	97	-	3	-	-
6:1 + DFO (%)		95	-	5	-
6:1 + BIPY (%)	92	8	-	-	-

Protein at a concentration of 2.5 mg/ml was mixed with ammonium iron (II) sulfate, (NH_4_)_2_Fe(SO_4_)_2_·6H_2_O. The final ammonium iron (II) sulfate concentration range applied was 0.35–1.75 mM, which corresponded to the Fe^2+^: protein molar ratios of 2:1, 4:1, 5:1, 6:1, and 10:1. First DLS measurement was recorded after approximately 5 min of incubation (at room temperature). DLS profiles were recorded every 5 min for 2 h. The Table shows data only for the 6:1 iron-to-protein ratio. Further details are described in the Methods section.

A different effect was observed at the iron-to-protein ratio of 10:1. In this case, after 60 min of incubation no observable monomers remained in solution (Fig 2B, blue). The major fraction of the oligomers (88%) consisted of particles with a mean hydrodynamic radius of about 5 nm, while a smaller fraction (12%) contained large particles with a diameter of up to 18 nm.

New measurements of all samples after 24 h of storage showed that the oligomerization reverted with time and only monomers remained in the solution (data not shown). In addition, in the 10:1 sample a small pellet of red-colored precipitate was clearly seen. This is consistent with earlier data, which showed that in contrast to assembled human frataxin, the short FXN^78-210^ variant was unable to keep oxidized iron for longer periods of time [10].

Therapies using iron chelators have been suggested for the treatment of FRDA. Among those is deferiprone, a bidentate ferric iron chelator that forms a 3:1 complex with iron, and DFO (deferoxamine, desferrioxamine, desferal), a bacterial siderophore produced by *Streptomyces pilosus* [39, 49, 50]. Here we tested the effect of the chelators on frataxin oligomerization. The addition of deferiprone at the iron-to-protein ratio of 6:1 to the FXN^81-210^ sample triggered the formation of a small percentage (3%) of oligomers with the hydrodynamic radius in the range of 8.5–14 nm (Table 1). These particles are larger than the particles formed in the absence of deferiprone (6–8.5 nm). However, after 24 h the 8.5–14 nm peak disappeared, and instead new smaller oligomers with a hydrodynamic radius in the range of 2.2–2.9 nm were formed (not shown). This suggests that the presence of the chelator contributes to oligomer stabilization since in the absence of chelator, as mentioned above, after 24 h no oligomers remained in the sample.

A more dramatic effect was observed with the second chelator, DFO. About 15 min after its addition (30 min after the start of the incubation of the protein sample with iron), about 95% of the sample consisted of oligomers with a hydrodynamic radius in the range 6–8.5 nm, while the remaining 5% had a radius in the range 15–25 nm (5%). Essentially, no monomers could be detected in the solution. The addition of the ferrous iron chelator BIPY at any iron-to-protein ratio did not alter the distribution of the oligomers, clearly suggesting that FXN^81-210^ oligomers are primarily stabilized by Fe^3+^.

For additional verification of the effect of Fe^3+^ on FXN^81-210^ oligomerization, we run DLS measurements using ferric iron solutions (FeCl_3_) prepared as described by Yoon & Cowan [13] (see also legend to Table 2). As shown in Table 2, a small number of medium-size oligomers formed at the iron-to-protein ratio of 6:1, however, most of the sample (97%) was still in a monomeric form. In contrast, at the iron-to-protein ratio of 8:1 essentially no monomers remained in the solution—84% formed particles with a hydrodynamic radius in the range of 6–8.5 nm, and the rest in the 15–25 nm range. At 10:1 iron-to-protein ratio the size of the majority of the particles shifted to the range 8.5–14 nm (87%), with the rest forming some larger structures. This clearly indicates that Fe^3+^ binds to the protein and contributes to particle stabilization. The results also indicate that initial occupation of all 6 binding sites, suggested by Yoon & Cowan [13], may be required for the formation of higher order oligomers.

**Table 2:** The effect of ferric iron on the oligomerization of human frataxin FXN^81-210^.

Hydrodynamic radius (nm)	1.6–3	6–8.5	8.5–14	15–25	< 30
6:1 (Fe^3+^: FXN^81-210^, %)	97	-	3	-	-
8:1 (Fe^3+^: FXN^81-210^, %)	-	84.4		15.6	-
10:1 (Fe^3+^: FXN^81-210^, %)			86.8	-	13.2

Solutions for measuring Fe^3+^—dependent oligomerization were prepared as described by Yoon & Cowan [13]: Ferric ion solutions were prepared by adding 0.5 M FeCl3 in 0.05 M HCl into 100 mM pH 7.5 HEPES buffer to obtain a desirable range of ferric ion concentration. To avoid solubility problems for ferric ion, a concentrated stock was prepared in acidic solution. DLS measurements were performed as described in the legend to Table 1 and in Methods section.

### Iron-induced oligomerization of *E*. *coli* CyaY and the effect of chelators studied using DLS

**Fig 3:**
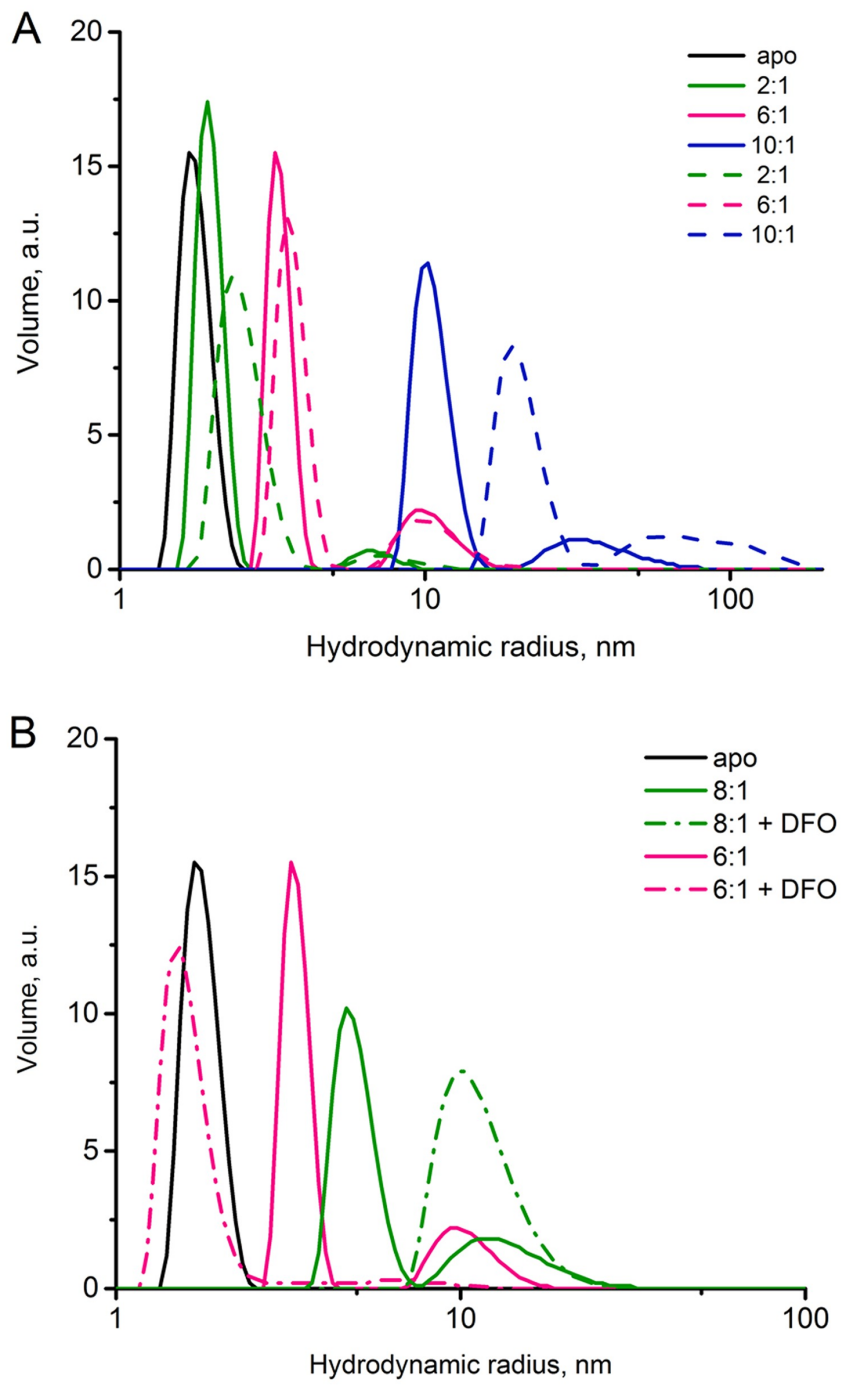
DLS studies of iron-dependent oligomerization of *E*. *coli* CyaY. **A**) CyaY was incubated for 1 h at 2:1 iron-to-protein ratio (green); 6:1, magenta; 10:1, blue. Dashed lines show the same sample after 26 h of incubation. In black, monomeric CyaY without addition of iron. On the x-axis the hydrodynamic radius and on the y-axis the volume-percentage of particles are shown. **B**) CyaY oligomerization with and without DFO. The protein was incubated at 6:1 (red) and 8:1 (green) iron equivalents; in black the protein without iron addition; dashed line show the sample after the addition of DFO.

As noted in the introduction, both CyaY and FXN^81-210^ share a common feature, which is the absence of the long N-terminal extension. This extension plays a crucial role in the stabilization of yeast and human frataxin FXN^42-210^ and FXN^56-210^ oligomers, suggesting that CyaY and FXN^81-210^ might use other mechanisms for oligomer stabilization. Although before the start of this work it was known that CyaY forms oligomers in the presence of iron [28], no studies comparing the oligomerization of CyaY and FXN^81-210^ have been published. Here, we study CyaY oligomerization in conditions similar to those used for FXN^81-210^. The results show that the addition of iron at iron-to-protein ratios of 1:1, 2:1, and 4:1 induced the formation of CyaY oligomers with a hydrodynamic radius in the range of 3–6 nm, even though monomeric CyaY was still the predominant form. However, in contrast to FXN^81-210^, for which monomers where depleted from solution at the iron-to-protein ratio of 10:1 (see above), CyaY monomers already at the iron-to-protein ratio of 6:1 could not be detected (Table 3 and Fig 3A). Instead, the sample contained a mixture of two particle pools with the hydrodynamic radius of 3.0–5.9 nm (77%) and 8.5–14 nm (23%, Table 3).

**Table 3:** Average size and distribution of particles of CyaY obtained from DLS measurements and the effect of iron chelators.

Hydrodynamic radius (nm)	1.6–2.1	2.2–2.9	3.0–5.9	6.0–8.5	8.5–14
CyaY Apo (%)	100	-	-	-	-
6:1 (Fe^2+^: CyaY, %)	-	-	77	-	23
6:1 + deferiprone (%)	-	95	-	-	5
6:1 + DFO (%)	93	-	-	7	-
6:1 + BIPY (%)	76	-	-	24	-

CyaY at a concentration of 2 mg/ml (0.16 mM) was mixed with ammonium iron (II) sulfate, (NH_4_)_2_Fe(SO_4_)_2_·6H_2_O in concentration range of 0.16 mM–1.64 mM. The Fe^2+^: protein molar ratios used were 1:1, 2:1, 4:1, 6:1, 8:1, and 10:1. Protein and iron were incubated at 30°C prior to DLS measurements. DLS profiles were recorded after 1 h of incubation. Further details can be found in the Methods section. Table shows data only for the 6:1 iron-to-protein ratio.

Increasing the iron-to protein ratio to 8:1 resulted in a further diversification of the oligomeric pools, with about 74% of the particles having a hydrodynamic radius in the range of 6–8.5 nm, and two additional fractions having their hydrodynamic radii within the ranges of 8.5–14 nm and >100 nm (25% and 1%, respectively, not shown). At the iron-to-protein ratio of 10:1, the sample contained only larger particles with a hydrodynamic radius in the ranges of 8.5–14 nm (83%) and 26–40 nm (17%). After 24 h of incubation the oligomers were still present in the CyaY samples (Fig 3A, dashed lines), which is a substantial difference, compared to FXN^81-210^.

We also studied the effect of deferiprone and DFO on iron-induced oligomerization of CyaY (Table 3). At 6:1 iron-to-protein ratio the effect of the chelators on CyaY oligomers was largely similar to that observed earlier for yeast frataxin [26]. The addition of deferiprone resulted in a shift of the hydrodynamic radius of the largest fraction of the oligomers (3–5.9 nm, 77%), towards smaller particles (2.2–2.9 nm, 95%). Part of these smaller particles presumably originate from the dissociation of the large-size particles (8.5–15 nm, 23%), of which only 5% remained in solution. The addition of DFO at the same iron-to-protein ratio disassembled most of the oligomers, with the main component being monomeric (93%) and the remaining fraction consisting of oligomers with the hydrodynamic radius in the range of 6–8.5 nm (7%, Fig 3B and Table 3). Interestingly, at the iron-to-protein ratio of 8:1 and in the presence of DFO the smallest and the largest peaks disappeared, and a single peak corresponding to the medium-size particles found in the initial experiments (8.5–14 nm, 100%, Fig 3B) was formed instead.

To assess the possible role of ferrous iron in the stability of the oligomers we also incubated CyaY oligomers obtained at the iron-to-protein ratio of 6:1 with the ferrous iron chelator BIPY. As seen in Table 3, the addition of BIPY also resulted in the dissociation of a large proportion of the oligomers (76% monomers), with the remaining 24% of the particles having a hydrodynamic radius in the range 6–8.5 nm. These results are consistent with earlier observations that showed the ability of Fe^2+^ to stabilize CyaY oligomers [28].

It should be noted that when any of the chelators was added before or simultaneously with iron, no oligomers were formed for either human or bacterial frataxin, which is presumably a result of a higher iron-binding affinity of the chelators as compared to the iron binding affinity of the proteins.

### Iron-induced FXN^81-210^ oligomers could not be detected using size-exclusion chromatography

**Fig 4:**
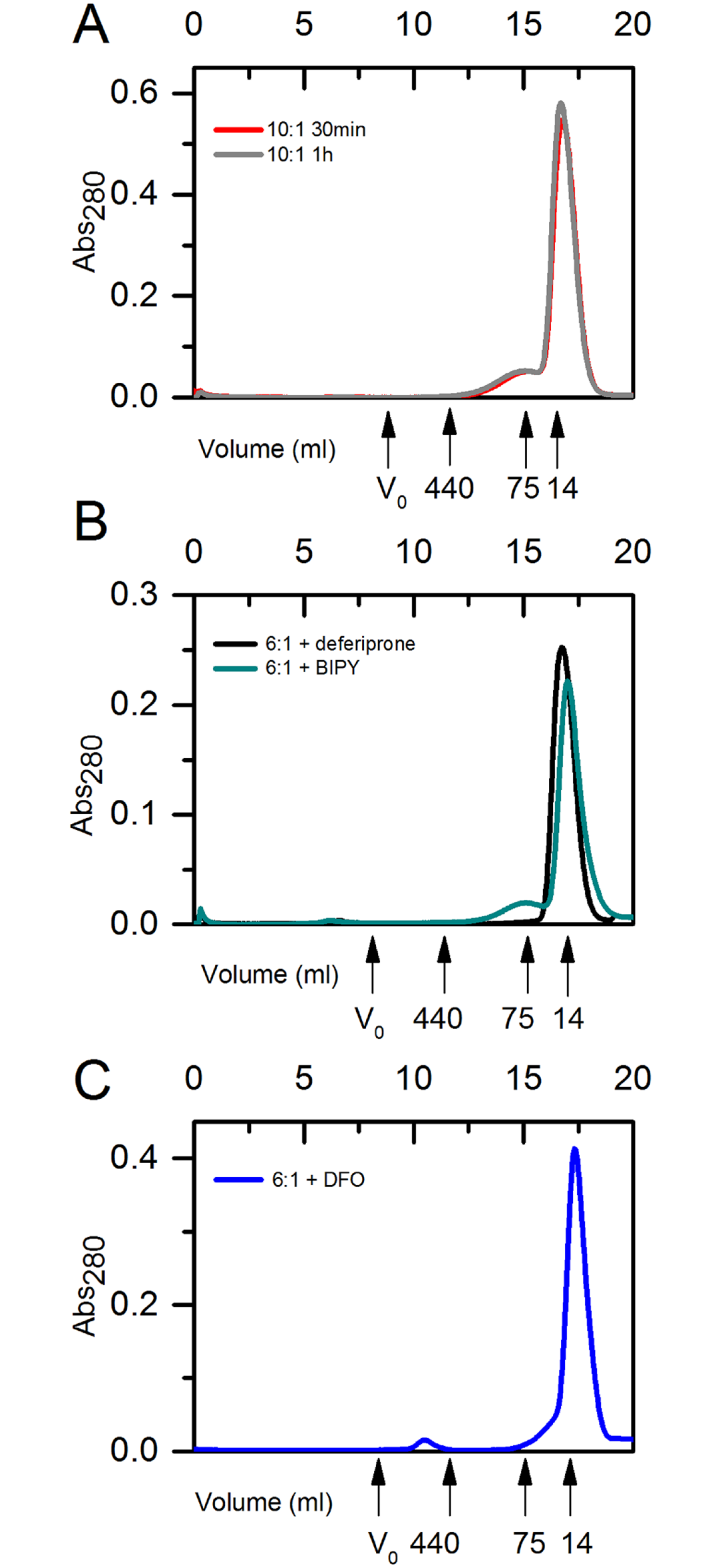
Size exclusion chromatography of human FXN^81-210^ incubated with iron. **A**) FXN^81-210^ incubated with 10:1 molar ratio of iron-to-protein for 30 minutes (red) and 1 h (dark gray). The main peak corresponds to monomeric FXN^81-210^, while the second peak corresponds to oligomeric protein in the tetrameric to hexameric size range of 50–80 kDa, **B**) FXN^81-210^ incubated with 6:1 molar ratio of iron-to-protein and 3 x excess of BIPY (green) and deferiprone (black). For the sample with deferiprone only one peak, at the size of monomeric FXN^81-210^, can be seen. For the sample with BIPY a main peak is at the size of monomeric FXN^81-210^, while the second broader peak is at the size of oligomers in the tetrameric-hexameric range of 50–80 kDa **C**) FXN^81-210^ incubated with 6:1 molar ratio of iron-to-protein and 3x excess DFO (blue). The main peak, corresponds to monomeric FXN^81-210^, 2 small peaks correspond to approximately ~75 kDa and 440 kDa.

Size-exclusion chromatography (SEC) has been the main method for studying iron-induced oligomerization of human frataxin [8, 48, 51]. Therefore, we also used this technique to try to obtain independent evidence for the complexes detected by DLS. Here FXN^81-210^ was incubated for 30 min and 60 min at the iron-to-protein ratio of 10:1 before loading the samples onto a Superdex 200 gel filtration column. The main component after 30 min of incubation was monomeric FXN^81-210^, and similar to the DLS results, about 10% appeared to be larger species eluting at the approximate molecular weight around 75 kDa (Fig 4A). The gel filtration profile of the sample incubated for 60 min was similar to that obtained after 30 min incubation. This is in contrast to the DLS studies, which at an iron-to-protein ratio of 10:1 after 60 min of incubation showed only oligomeric particles (Fig 2). This indicates that a large fraction of the oligomers had disassembled during gel filtration.

These results confirm earlier results and demonstrate that SEC should not be a method of choice for assessing oligomer formation in the case of human frataxin. However, in the case of Yfh1 oligomers, SEC could still be used to observe oligomeric species, which apparently had higher stability than those of FXN^81-210^ [26]. We also attempted to run SEC in the presence of iron, however, in this case the column was quickly plugged, probably as a result of the presence of iron oxides, and the experiment had to be terminated.

Interestingly, FXN^81-210^ oligomers formed after the addition of deferiprone were also unstable, with a dominating monomeric protein peak visible after gel filtration (Fig 4B). In the presence of DFO the main fraction still eluted as monomers, followed by a broad peak with apparent molecular mass of around 75 kDa, and a small peak eluting just after the void volume (Fig 4C). The 75 kD peak was also present when BIPY was incubated with the sample (Fig 4B).

### Visualization of the iron-induced FXN^81-210^ oligomers with transmission electron microscopy

**Fig 5:**
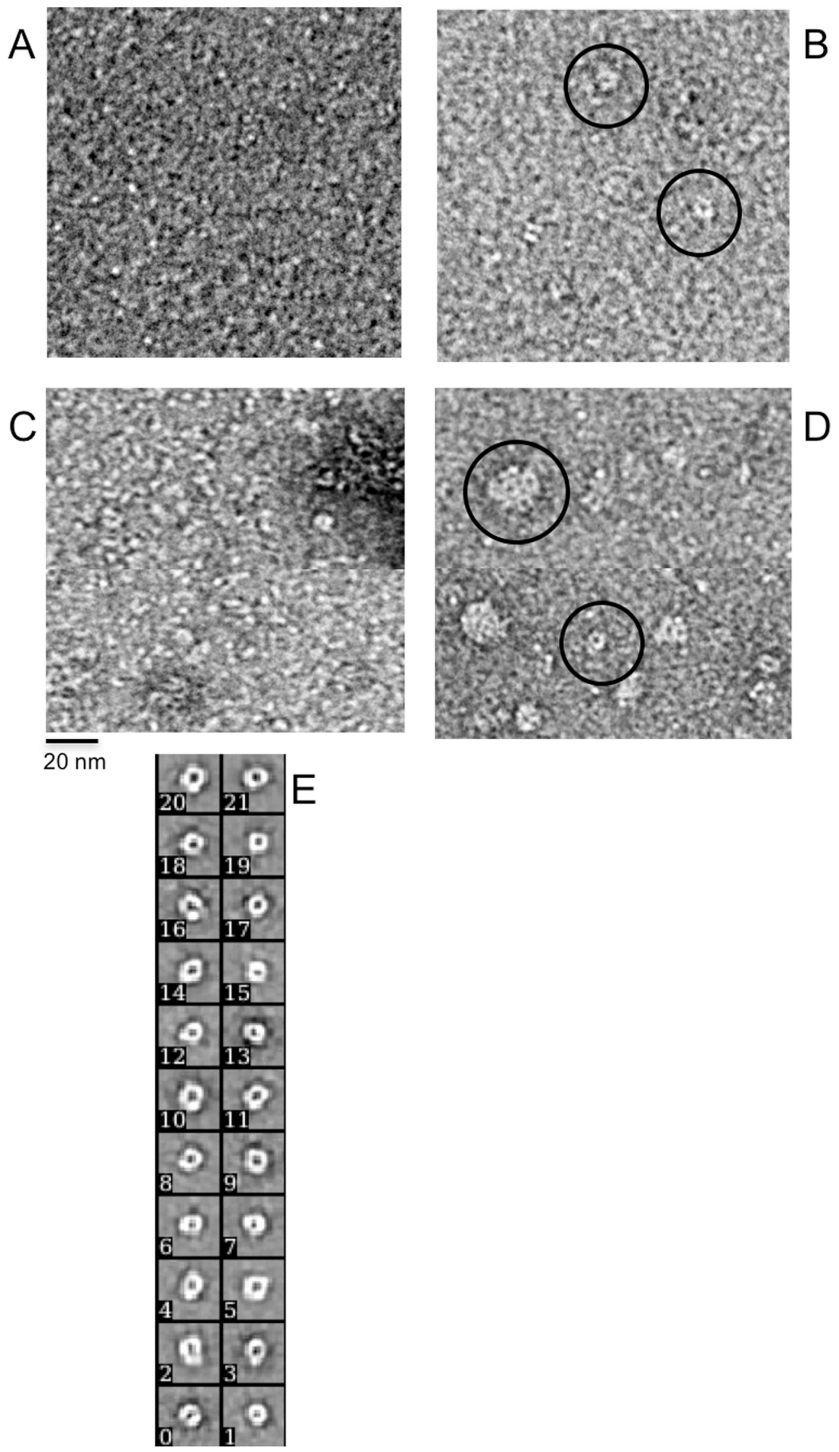
Negative staining TEM images of iron-induced FXN^81-210^ oligomers. **A**) Monomeric FXN^81-210^. **B)** FXN^81-210^ after 30 min of incubation with iron at 6:1 of iron-to-protein molar ratio. Ring-shaped particles are marked by black circles. **C**) FXN^81-210^ incubated with iron at 6:1 molar ratio of iron-to-protein and with deferiprone added. **D**) FXN^81-210^ incubated with iron at 6:1 molar ratio of iron-to-protein and with DFO added. Ring-shaped particles and larger spherical particles are marked by black circles. **E**) Class averages of the ring-shaped structures obtained from 1265 particles. The tetrameric character of the ring-structures can be clearly seen on some of the class averages. The EM images have a magnification of 55,000x.

The DLS results were confirmed using negatively stained EM images. Fig 5A shows that in the absence of iron, no FXN^81-210^ aggregates can be found on the grids. At the iron-to-protein ratio of 6:1 a small number of particles, which appear to have ring-shaped form, can be distinguished (Fig 5B). These particles presumably correspond to those seen in the DLS experiment (Table 2), which had a hydrodynamic radius in the range of 6–8.5 nm. The addition of deferiprone promoted the formation of a larger number of ring-shaped structures, but also irregular aggregates (Fig 5C). A significantly higher number of ordered ring-shaped structures were found in the presence of DFO (Fig 5D). In addition, spherical particles with a diameter in the range of 20–25 nm were visible on the grids (Fig 5D).

For a better characterization of the ring-shaped particles, we used the method of single particle reconstruction (Fig 5E). The tetrameric character of the particles is clearly visible for some of the class-averages shown on the figure, which suggests an arrangement of monomers similar to that proposed earlier for CyaY [30]. Due to the preference of the ring-shaped structures to land ringside up, few side views could be found on the micrographs, making proper 3D reconstruction impossible without image tilt. The variations in the outer diameter of the rings, as determined from the micrographs by the program Digital Micrograph, was between 8–11 nm, with a preference for 8 nm, while the diameter of the central hole was estimated to be 3–4 nm. This correlates well with the dimensions obtained from class averages and reflects the polydispersity seen in the DLS measurements.

The estimated thickness of 2–3 nm fits well with the size of the crystal structure of monomeric human frataxin, taking into account the commonly known flattening effect caused by negative staining. With these constraints it is most likely that the structures are built up by one layer of monomers, as a double-layer structure would have a thickness of at least 4 nm.

### Arrangement of subunits studied by crosslinking MS

**Fig 6:**
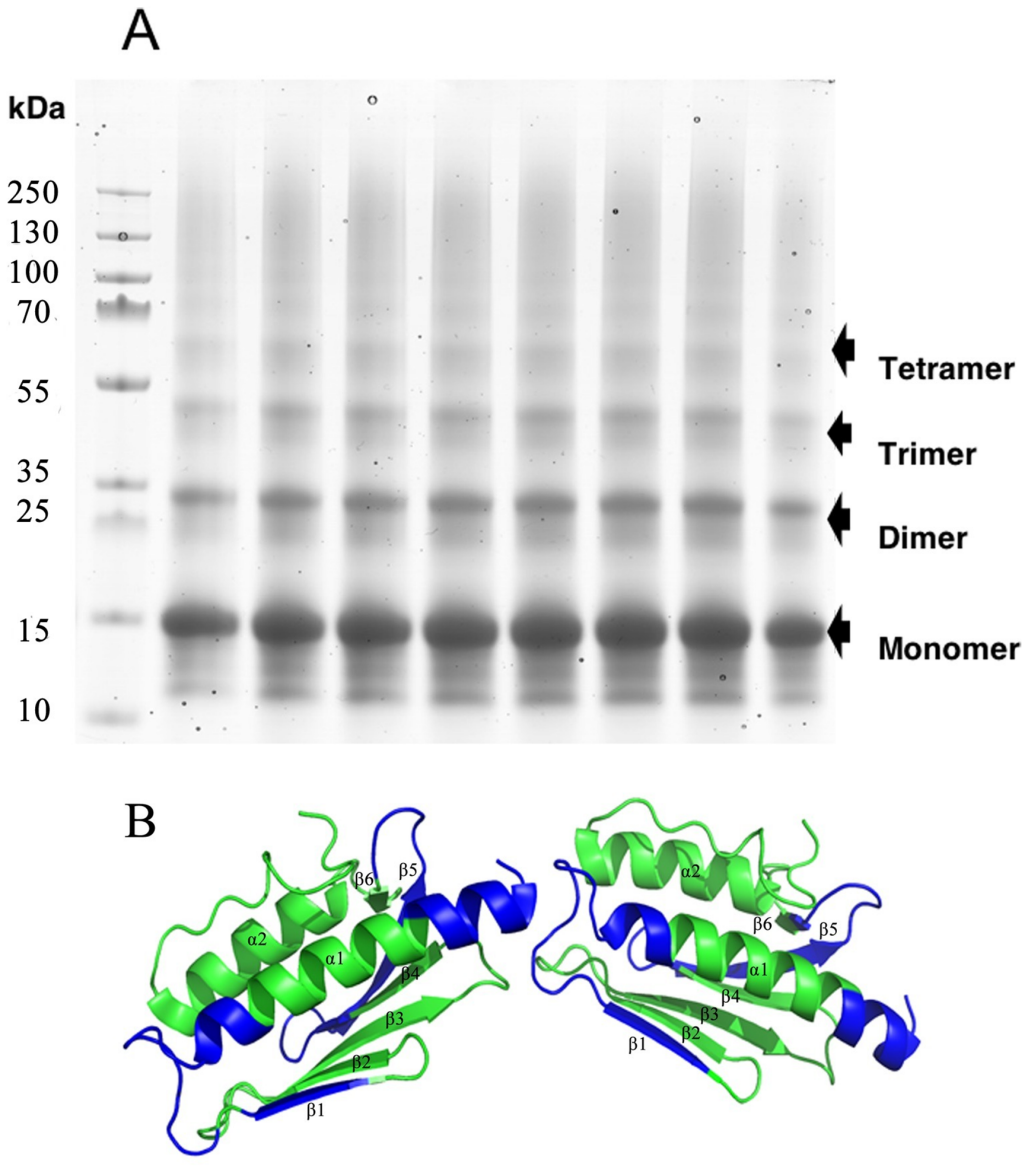
Crosslinking of iron- and hydrogen peroxide-induced FXN^81-210^ oligomers. **A**) SDS-PAGE gel of crosslinked FXN^81-210^ incubated with iron (II) at a 6:1 molar ratio of iron-to-protein and hydrogen peroxide with bands of monomeric, dimeric, trimeric, and tetrameric size indicated. **B**) Crystallographic structure of human frataxin (PDB entry 1EKG) was used here to generate a head-to-tail dimer. The regions of the structure shown in [33] to have reduced deuterium incorporation after Fe^3+^ binding are highlighted.

To obtain experimental evidence in support of a possible arrangement of the monomers within the FXN^81-210^ oligomers, we used cross-linking mass spectrometry with a lysine-specific crosslinker. The crosslinked samples were subsequently subjected to SDS-PAGE, and bands representing dimers, trimers, and tetramers (Fig 6A) were excised, digested with trypsin and subjected to LC-MS/MS. Data were analyzed with the software *MassAI* to detect the crosslinked peptides (Table A, with approved MS/MS-spectra in the S1 File). The length of the crosslinker allows amino acids with a Cα-Cα distance of up to 30 Å to be crosslinked [52]. Such crosslinks are likely to be intra-monomeric, but in some cases, they could also be inter-monomeric. On the other hand, crosslinks with distances exceeding 30 Å are most likely to be inter-monomeric. The analysis of trimer and tetramer bands on the SDS-PAGE (Fig 6A), revealed similar inter-monomeric crosslinks. These crosslinks, K152-K195 at 33.2 Å, K152-K197 at 30.9 Å, and K171-K195 at 29.2 Å, support a head-to-tail arrangement of monomers (Fig 6B). In this mode the N-terminus (residue 81–96) and the connecting loop between strands β3 and β4 (residue 148–153) as well as the loop between strands β5 and β6 (residue 167–172) of one monomer are facing the end of helix α1 and the connecting loop to strand β1 (residue 111–124) as well as the loop between strands β2 and β3 (residue 135–142) and the end of helix α2 (residue 192–196).

Since the first amino acid residue in the human frataxin PDB structure (1EKG) is L90, the distance from the primary amine of the amino terminal S81 to the lysine residues crosslinked to it could not be measured directly. An estimate of these distances was made by adding to, or subtracting from the distance from L90 to the crosslinked residue in the X-ray structure (PDB entry 1EKG), the estimated length of the peptide containing the missing 9 residues (Table A in the S1 File). These values provide the maximum and minimum possible distances between the cross-linked residues. For clarity, the sequence of human FXN^81-120^ (sp|Q16595|81–210) is shown below:

S_81_GTLGHPGSL_90_DETTYERLAEETLDSLAEFFEDLADKPYTFEDYDVSFGSGVLTVKLGGLGTYVINKQTPNKQIWLSSPSSGPKRYDWTGKNWVYSHDGVSLHELLAAELTKALKTKLDLSSLAYSGKDA

Unfortunately, the low conservation level and high flexibility of the N-terminal part of frataxin does not allow the use of the known structure of the same region of yeast frataxin for an accurate estimation of the distances between the cross-linked residues.

**Fig 7:**
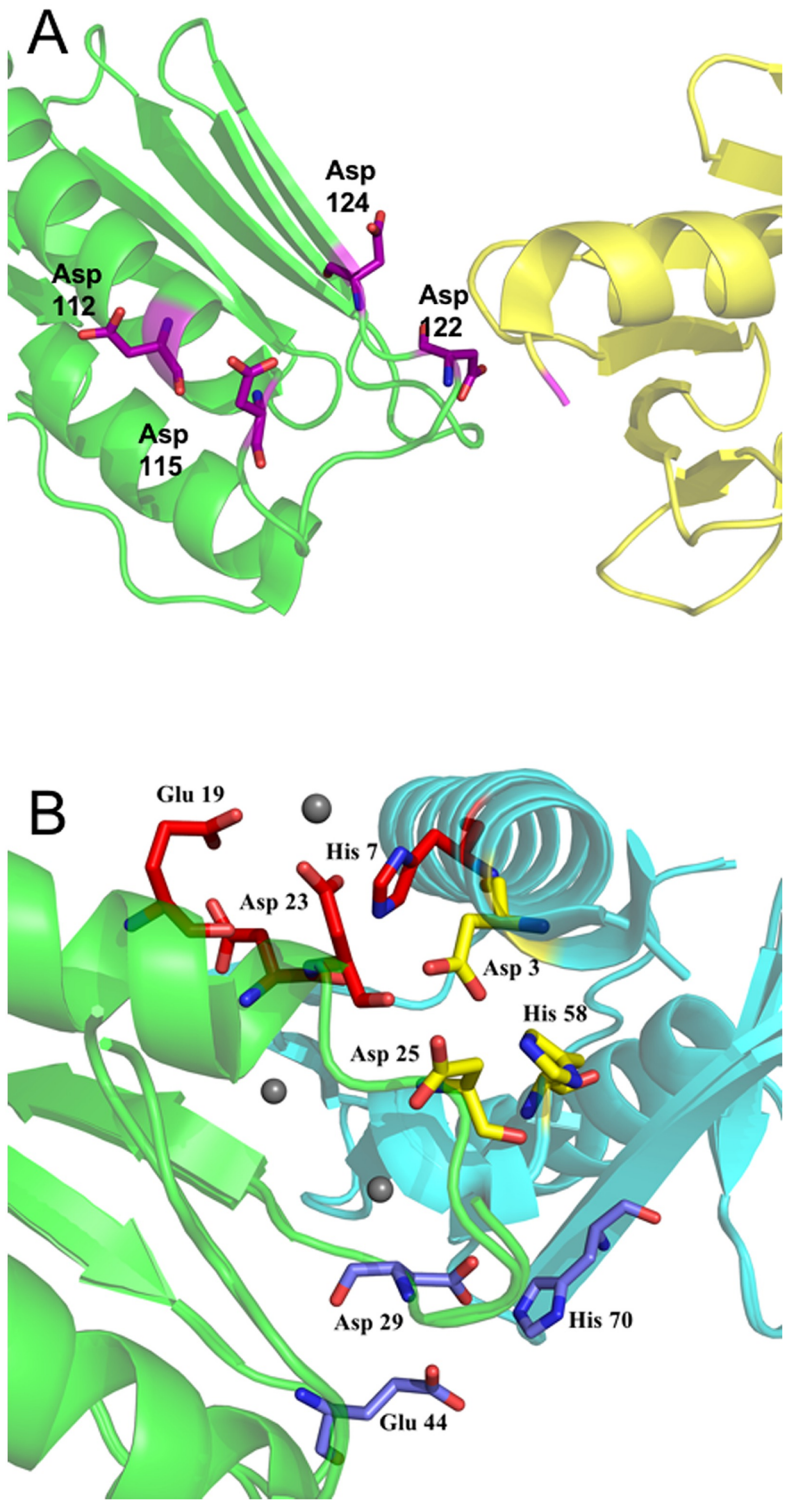
The interface between subunits in the head-to-tail arrangement. **A**) Human frataxin interface with residues D112, D115, D122, and D124 marked in magenta. The N-terminus is colored in pink and the different monomers within the dimer are shown in yellow (head) and green (tail). A crystal structure (PDB entry 1EKG) was used for preparing the figure. **B**) CyaY interface and the residues making up the potential metal binding sites are shown. Residues H7, E19, D22, and D23, which may build up the first metal binding site, are shown as red sticks; D3, H58, and D25 may participate in the second site (yellow sticks); and H70, D29, and E44 (blue sticks) in the third. Gray spheres show Europium ions bound in the 2P1X crystal structure. Residues involved in metal binding in the crystal structures of CyaY in complex with Co and/or Eu are labeled. The different monomers in the dimer are shown in green (head) and blue (tail). Crystal structures (PDB entry 2P1X, 2EFF, and 1EW4) were used in the preparation of the figue.

The crosslinks obtained for *E*. *coli* CyaY gave the opportunity to re-evaluate an earlier model based on SAXS and docking experiments [30]. Since CyaY contains fewer lysine residues than FXN^81-210^, only one crosslink could be detected, which was between the primary amine on the N-terminal amino acid (M1) and K65 (see MS/MS-spectrum in the Supporting Information). This crosslink also supports a head-to-tail arrangement of the CyaY monomers. Models of FXN^81-210^ and CyaY dimers showing the possible stabilizing interactions in the head-to-tail arrangement are shown on Fig 7A and 7B.

The models reveal that for both proteins, the residues found earlier to be involved in metal binding in the crystal structures with bound metals or shown to be affected by iron binding in NMR experiments, are located at the interface between the monomers [31, 33, 51, 53]. For FXN^81-210^, at least one potential metal binding site can be distinguished at the interface between the monomers. In this site H86 from the N-terminus of one monomer (not shown on the figure, PDB entry starts at residue 90) may bind iron together with D112 and D115 located at the end of the first α-helix of the second monomer or possibly D122 and D124 on the first β-strand, which follows the α-helix. This region of the structure (marked in blue in Fig 6B) has been proposed to be involved in iron binding in the work by Gentry et al. [33].

For CyaY, three possible iron binding sites can be distinguished at the interface. The first site includes H7 from one subunit and E19, D22, and D23 from the second. Residues E19 and D23 have been found to interact with Eu^3+^ in a crystal structure [53]. The second site includes resides D3 and H58 from one subunit and D25 from the other. Two of these residues, D3 and H58 together with D55 have been found to bind Co^2+^ and Eu^3+^ in the crystal structure. The third potential metal-binding site may involve H70 from one subunit and D29 and E44 from the second. It should be noted that solvent molecules could also complement the binding sites. The higher number of acidic residues at the interface between CyaY monomers compared to the FXN^81-210^ monomer interface probably provides an explanation for the higher stability of CyaY oligomers, as compared to FXN^81-210^ oligomers.

## Discussion

In this work, we show that iron binding to FXN^81-210^ and CyaY is intimately linked to oligomerization, and compare the oligomerization of human FXN^81-210^ and bacterial CyaY frataxin. Using DLS and SAXS measurements, we show that at iron-to-protein ratios higher than 2:1 the structures formed by CyaY are larger than those formed by FXN^81-210^. In addition, the data suggest that the total of 6 iron atoms/monomer are required for full transition of CyaY to the oligomeric form. It cannot be excluded that at this stage ferrihydrite micro-crystallites may start to form, supporting the stability of higher order oligomeric states—from 3 nm particles to 6–8.5 nm and even larger, reaching the 8.5–14 nm and 26–40 nm ranges. On the other hand, FXN^81-210^ with its labile iron binding properties [8], requires higher iron-to-protein ratios (10:1) before it can form large oligomers. It has been noted earlier that the iron-to-protein ratio of 10:1 is close to the iron binding capacity limit of human frataxin [5, 13], which again suggests that only after reaching this limit larger particles start to be stable. In conditions of normal iron content in cells the iron bound to FXN^81-210^ will probably never reach that critical level, which would prevent the formation of larger structures. This might explain the inability of short variants of human frataxin to form large particles with a ferrihydrite mineral core similar to that formed by the longer isoforms [10, 11].

The results also suggest that the number of acidic residues at the monomer-monomer interface may control the mode of oligomerisation of the short-length frataxin forms, and presumably even iron mineralization. These acidic residues are part of an acidic cluster, conserved in all frataxin structures. The number of residues involved in the cluster in different species is also comparable– 13 in the human and yeast Yfh1 sequences and 11 in CyaY. Of these, in the human structure 8 residues belong to helix 1, in yeast 5 and in *E*. *coli* 7. However, alignment of the sequences shows that only 3 of the cluster residues have conserved positions across all species (not shown), while the positions of the others are variable. The higher number of acidic residues at the interface between CyaY monomers, as compared to FXN^81-210^, is a direct consequence of this position variability. This suggests that by varying the location of the acidic residues within helix 1 and the following loop and β-strand (Fig 7), organisms may control the mode of frataxin oligomerization.

The monomer-monomer contact area in CyaY may also provide the environment required for iron mineralization in a way similar to that described earlier for yeast frataxin [30]. In that case the corresponding iron mineralization sites were suggested to be located at the interface between trimers within higher-order oligomers. Presumably, iron mineralization is not one of the primary functions of human FXN^81-210^, rather FXN^56-210^, which has been suggested to catalyze the ferroxidation reaction, is also involved in mineralization [5, 10]. FXN^81-210^, on the other hand, may serve in temporary iron storage and delivery to biochemical processes [8, 17, 18]. Thus, Gakh et al. showed that while FXN^81-210^ could deliver F^3+^ to ISC assembly, the longer frataxin isoform FXN^42-210^ delivered both F^2+^ and F^3+^ [10]. A limited iron storage and oligomerization function of FXN^81-210^ may be used in conditions of iron overload, e.g. by forming larger oligomeric particles similar to those observed here. Such function may be of importance in Friedreich ataxia cells, in which FXN^42-210^ has been shown to be consistently more depleted than FXN^81-210^ [8]. These data suggest that while the same form of bacterial and yeast frataxin have evolved to deliver and store iron, human frataxin has followed a different strategy, in which different isoforms are combined to perform the functions of iron detoxification, storage and delivery. Currently it is not known if FXN^81-210^ oligomers observed here are of physiological relevance, since in normal cells longer frataxin isoforms and ferritin should be able to take care of surplus iron.

The metal binding role of the acidic residues, suggested here to contribute to monomer-monomer interactions, has been verified in several earlier studies. In a recent detailed work by Gentry et al. [33], UV-visible and NMR spectroscopy combined with backbone amide hydrogen/deuterium exchange mass spectrometry (HDX-MS) were used to study the stoichiometry of metal binding (iron and cobalt) to FXN^81-210^ and to characterize possible effects of metal binding on the structure of the protein [33]. The spectroscopic results showed that FXN^81-210^ binds three metal ions and that the highest-affinity binding site includes H86, located in the disordered N-terminal region. In addition, they also suggested that D112 and D115, which are conserved in most frataxin sequences, are also involved in metal binding. HDX-MS results also showed that metal binding leads to reduced deuterium incorporation in several parts of the structure, which include the N-terminus, helix 1, strand 1, and strands 4–5 (residues 81–98, 110–127, and 156–172, colored in Figs 6 & 7). Although the authors did not consider the possibility of the formation of dimers and higher-order oligomers by FXN^81-210^, a head-to-tail arrangement of the monomers may explain the reduced deuterium incorporation observed in the experiments. In this model, the iron binding site involving H86 may also involve D112 and D115, which are positioned at the end of helix 1. It should be noted that the iron-to-frataxin ratios used in this study were 2:1 and 3:1, which is in the same range as the ratios used in our studies.

Available experimental results clearly indicate that the mode of stabilization of short-form frataxin oligomers is different from that employed by FXN^42-210^, FXN^56-210^ and yeast frataxin, which require the long N-terminal extension for oligomer stabilization [5, 8, 10]. However, while FXN^42-210^ and FXN^56-210^ oligomers form independently of the presence of iron, yeast frataxin needs iron for oligomerization [5, 19, 26, 27, 29, 30]. This suggests that there are at least three different modes of frataxin oligomer stabilization. The first is stabilization only by the N-terminal extension (human FXN^42-210^ and FXN^56-210^). In the case of human frataxin FXN^42-210^, it has even been shown that the oligomerization was reversible—the oligomers can be dissociated into monomers in mild denaturing condition (in the presence of urea), and re-oligomerize after removal of the denaturant [54]. The second mode is Iron-induces stabilization promoted by the N-terminal extension (yeast frataxin Yfh1), and the third is iron-induced oligomerization of short forms of frataxin, which depends on iron binding to the acidic residues at the interface between monomers. It appears that oligomers of type 1, which are stabilized by the long N-terminal extension are more stable and may be larger in size, while type 3 oligomers are more labile/dynamic and smaller in size. On the other hand, the intermediate type 2 forms multiple oligomeric states, from a trimer to hexamers and other multiples of trimmers, up to a 24-mer [29, 30, 35, 36].

Iron overload is one of the consequences of frataxin deficiency in patients with FRDA, and therapies using iron chelators have been suggested for the treatment of the disease. Deferiprone, which we test here, is a bidentate ferric iron chelator that forms a 3:1 (chelator:iron) complex with iron with a high complex formation (K_f_) constant of 10^31^. Deferiprone has been used since the late 1980s in iron overload treatment and has also undergone limited clinical trials to evaluate its use in combination FRDA therapy [39, 49]. DFO, the second ferric iron chelator tested here, also known as deferoxamine, desferrioxamine, and desferal, is a bacterial siderophore produced by *Streptomyces pilosus*. Since the 1970s, the compound has been used in the treatment of iron overload, such as in β-thalassemia [49, 50]. DFO is a hexadentate hydrophilic chelator that forms a 1:1 octahedral complex with ferric iron under physiological conditions. However, since it does not penetrate the blood-brain barrier, it cannot be used in the treatment of neurodegenerative diseases [49, 55, 56].

Our results show for the first time that deferiprone and DFO promote FXN^81-210^ oligomerization, and that different chelators may have different effects on the oligomerization state, with DFO promoting the formation of a larger number of ordered structures. This finding may have important implications for the use of chelators in the treatment of FRDA, although at the present stage it is unclear how DFO and deferiprone stabilize FXN^81-210^ oligomers and whether this oligomerization has any effect at physiological conditions. One of the factors, which may contribute to the propensity of DFO and deferiprone to induce ordered structures could be related to their ability to bind at the interface between FXN^81-210^ monomers and stabilize subunit contacts. Further studies, including the development of an in vitro assay to assess the possible effects of chelators in cells, combined with tests of the effect of a number of other chelators, may shed some light on the mechanism of their action. This would also allow a structure-activity relationships model to be constructed, which would help in the design of new and more efficient chelators. Perhaps a combination of chelation and antioxidant functions in the same molecule would also help to attenuate the effects of reactive oxygen species in mitochondria [57].

## Supporting information

S1 File Table and Figures showing details of the MS experiments. (PDF)
