# Supplementary material for: Iron-induced oligomerization of human FXN81-210 and bacterial CyaY frataxin and the effect of iron chelators
Source: PLoS One. 2017 Dec 4;12(12):e0188937. doi: 10.1371/journal.pone.0188937 (PMC5714350; doi:10.1371/journal.pone.0188937)
Supplement: S1 File — (PDF) [file pone.0188937.s001.pdf]

Supplementary information

MSMS-spectra for 15 crosslinks detected within human frataxin,1 crosslink detected in *E. coli* frataxin homologue CyaY.

An example of output from the software MassAI is shown on next page.

Following pages show 16 MSMS-spectra directly exported as emf-files from the software with all annotations of ions retained and presented with transparent labels for convenient overview. Fragment-rich spectra require zooming in for full legibility.

Color coding in MSMS spectra:
y-ions (blue), b-ions (green), peptide A/B ions (purple), unexplained (red)

Table A. Crosslinking data for human FXN$^{81\text{-}210}$ after iron-induced oligomerization.

Number of crosslinked subunits	Crosslinked amino acid residues	Distance if crosslink is intra-monomeric C_α - C_α (Å)	Type of crosslink	Arrangement supported by inter-monomeric crosslink
dimer	S81-K152	-23.4	intra or inter	
	S81-K171	-19.6	intra or inter	
	S81-K192	2.8	intra or inter	
	S81-K195	6.1	intra or inter	
	S81-K197	2.4	intra or inter	
	K152- K195	33.2	inter	head-to-tail
	K152- K197	30.9	inter	head-to-tail
	K195- K197	6.9	intra or inter	
	K197- K164	22.8	intra or inter	
	K197- K197	-	inter	tail-to-tail
trimer	S81-K171	-19.6	intra or inter	
	S81-K192	2.8	intra or inter	
	S81-K197	2.4	intra or inter	
	K152- K195	33.2	inter	head-to-tail
	K152- K197	30.9	inter	head-to-tail
	K171- K197	24.3	intra or inter	
	K192- K195	5.1	intra or inter	
	K192- K197	8.1	intra or inter	
	K197- K164	22.8	intra or inter	
	K197- K195	6.9	intra or inter	
	K197- K197	-	inter	tail-to-tail

tetramer	S81-K152	-23.4	intra or inter	
	S81-K164	-11.1	intra or inter	
	S81-K195	6.1	intra or inter	
	S81-K197	2.4	intra or inter	
	K152- K164	13.6	intra or inter	
	K152- K195	33.2	inter	head-to-tail
	K152- K197	30.9	inter	head-to-tail
	K171- K195	29.2	inter	head-to-tail
	K197- K164	22.8	intra or inter	
	K197- K195	6.9	intra or inter	
	K197- K197	-	inter	tail-to-tail

EXAMPLE OF OUTPUT FROM SOFTWARE

K116-K114 = K197-K195

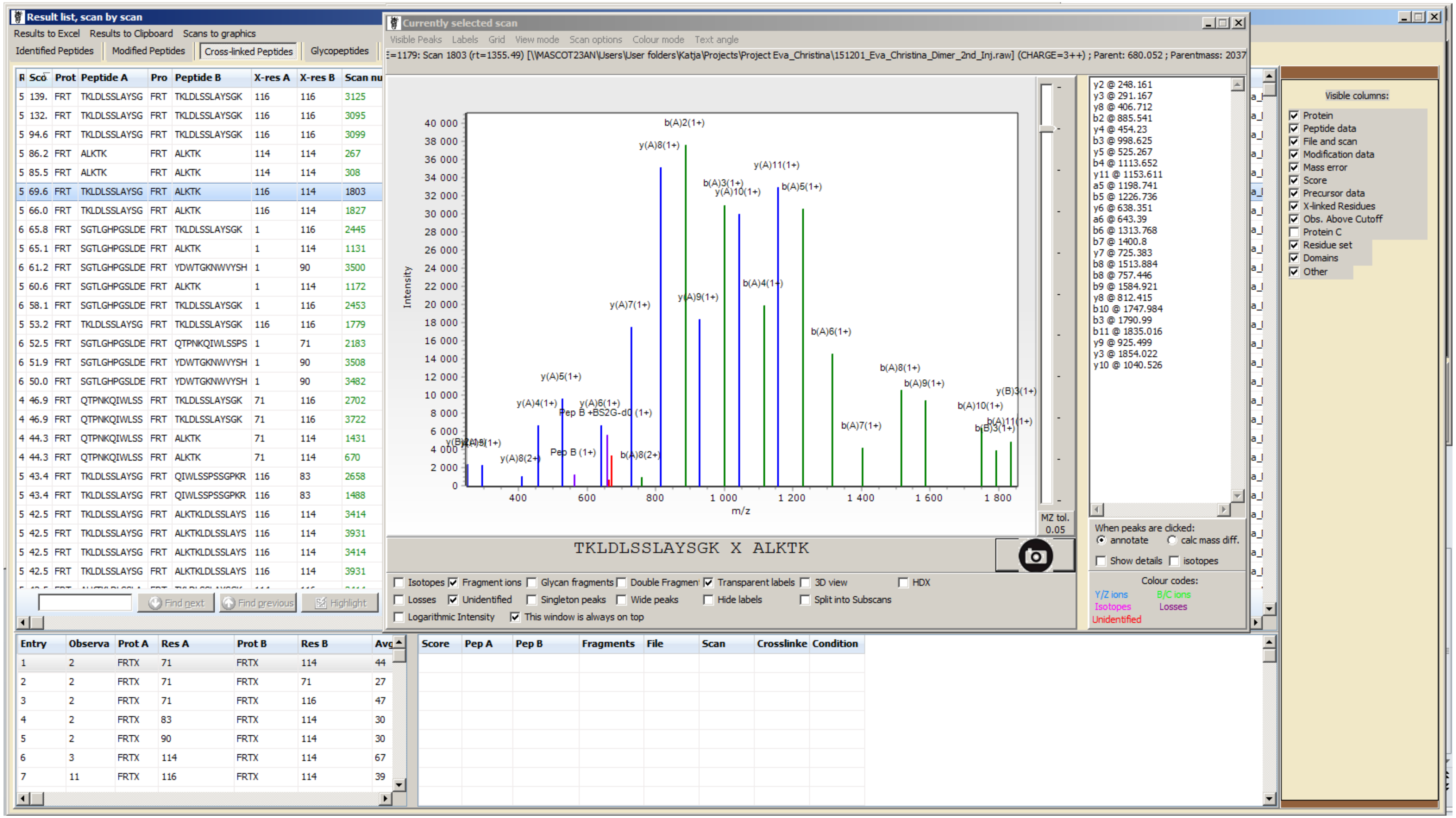

160218_FXN$^{81-210}$ Dimer_BS2G, K197-K195, TKLDLSSLAYSGK +ALKTK

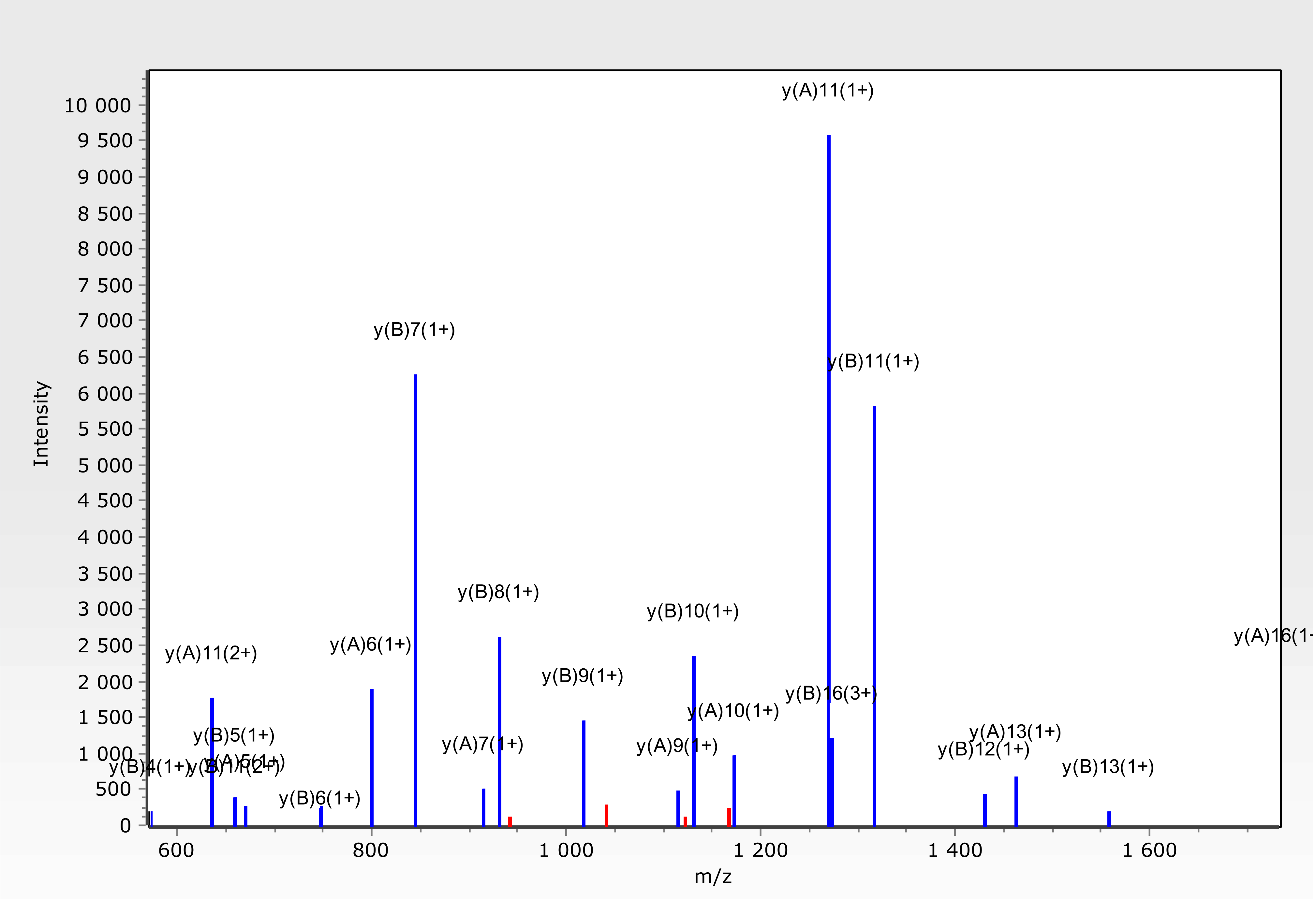

160218_ FXN$^{81\text{-}210}$ Dimer_BS2G, K81-K152, SGTLGHPGSLDETTYER + QTPNKQIWLSSPSSGPKR

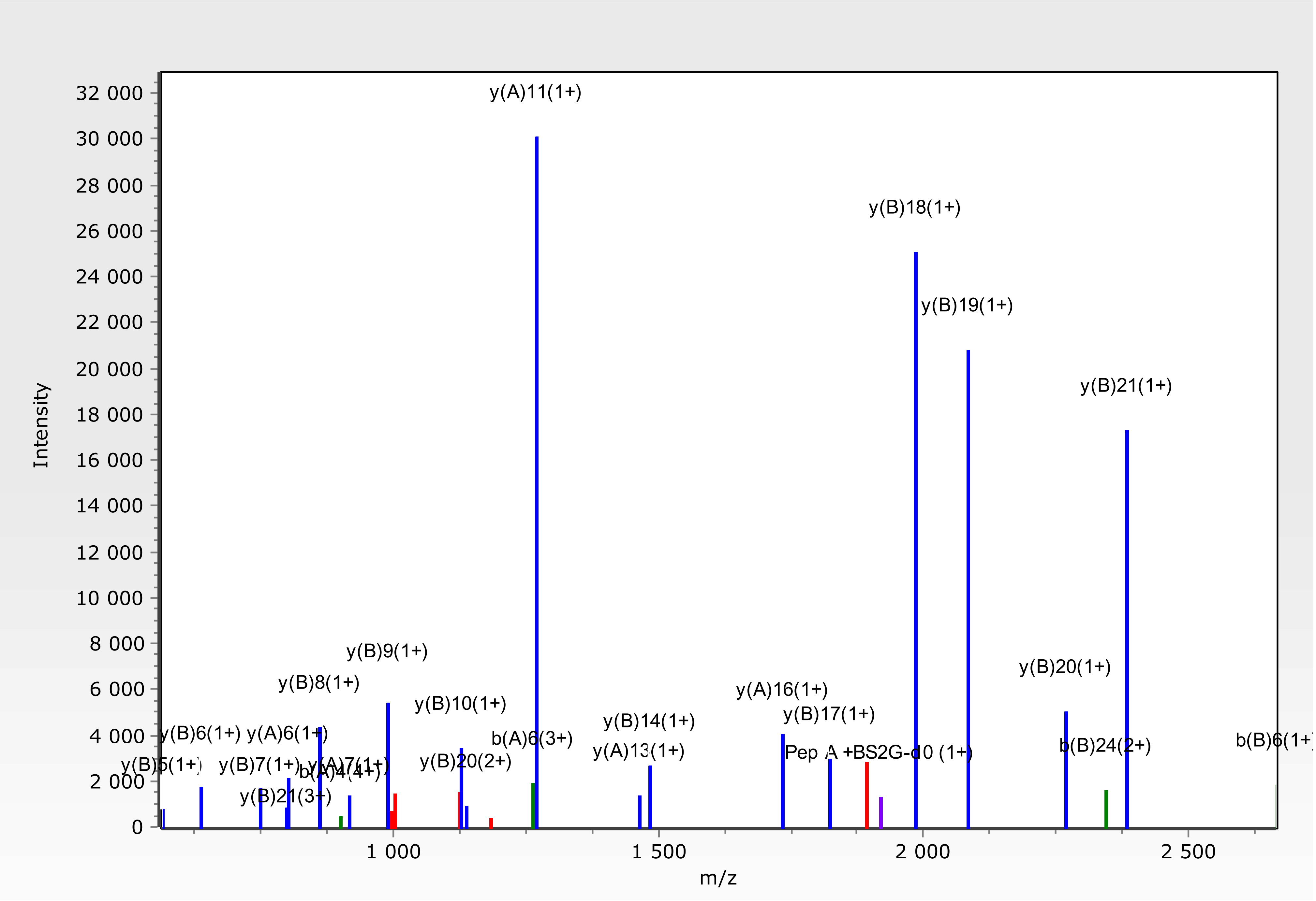

160218_ FXN$^{81-210}$ Dimer_BS2G, K81-K171, SGTLGHPGSLDETTYER + YDWTGKNWVYSHDGVSLHELLAAELTK

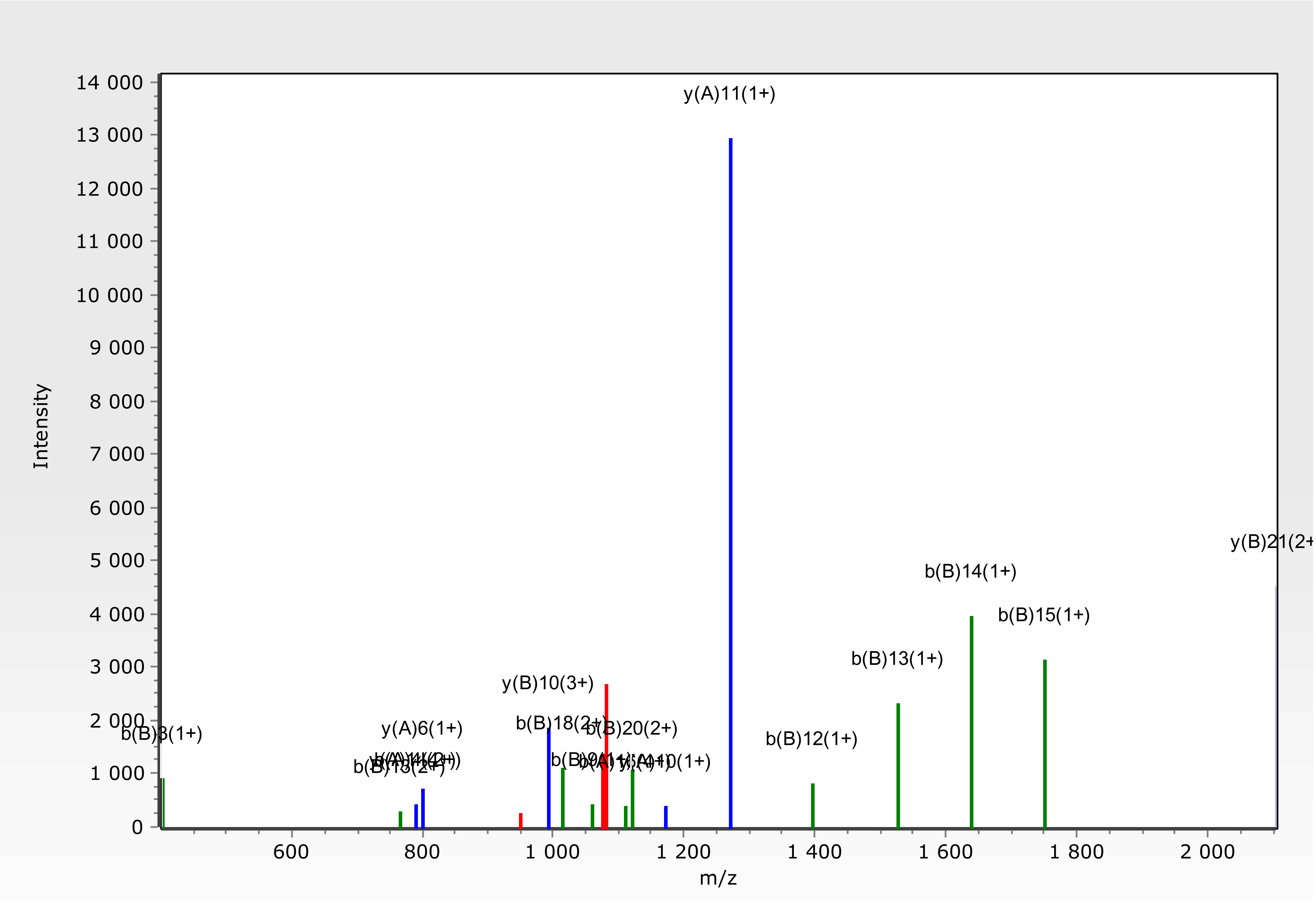

160218_ FXN$^{81-210}$ Dimer_BS2G, K81-K192, SGTLGHPGSLDETTYER + NWVYSHDGVSLHELLAAELTKALK

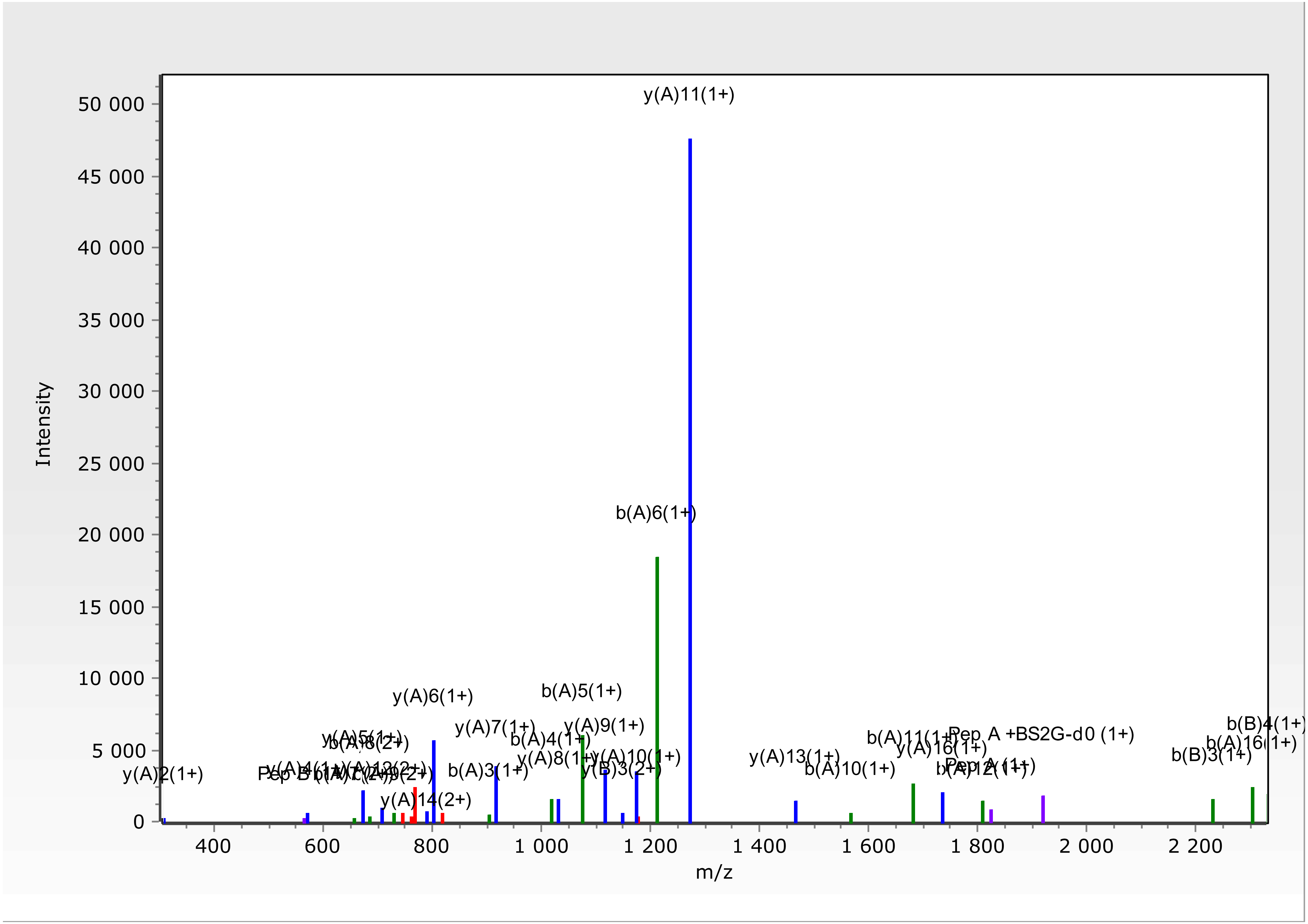

160218_ FXN$^{81-210}$ Dimer_BS2G, K81-K195, SGTLGHPGSLDETTYER + ALKTK

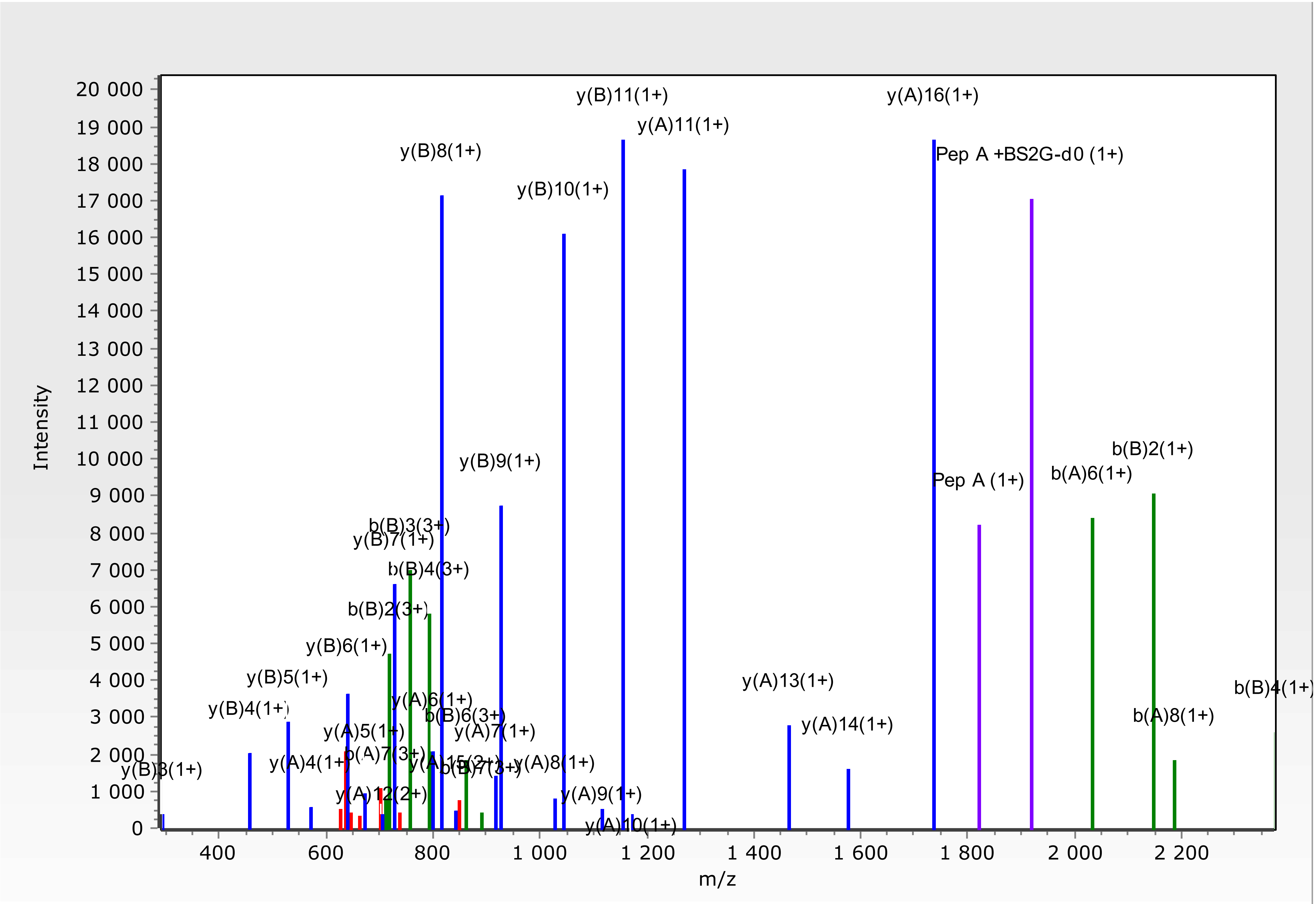

160218_ FXN$^{81-210}$ Dimer_BS2G, K152-K195, SGTLGHPGSLDETTYER + TKLDLSSLAYSGK

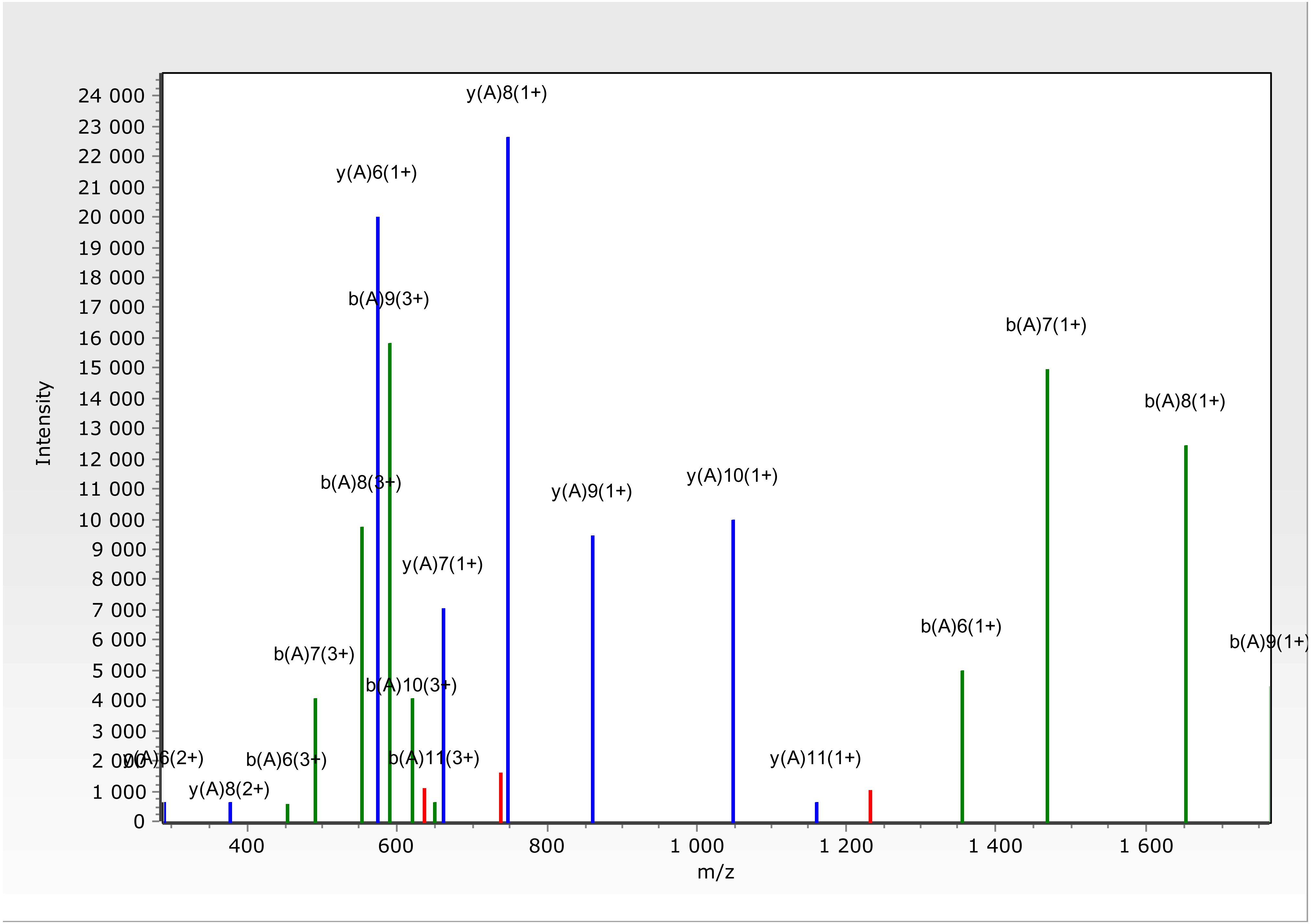

160218_ FXN$^{81-210}$ Dimer_BS2G, K152-K195, QTPNKQIWLSSPSSGPK + ALKTK

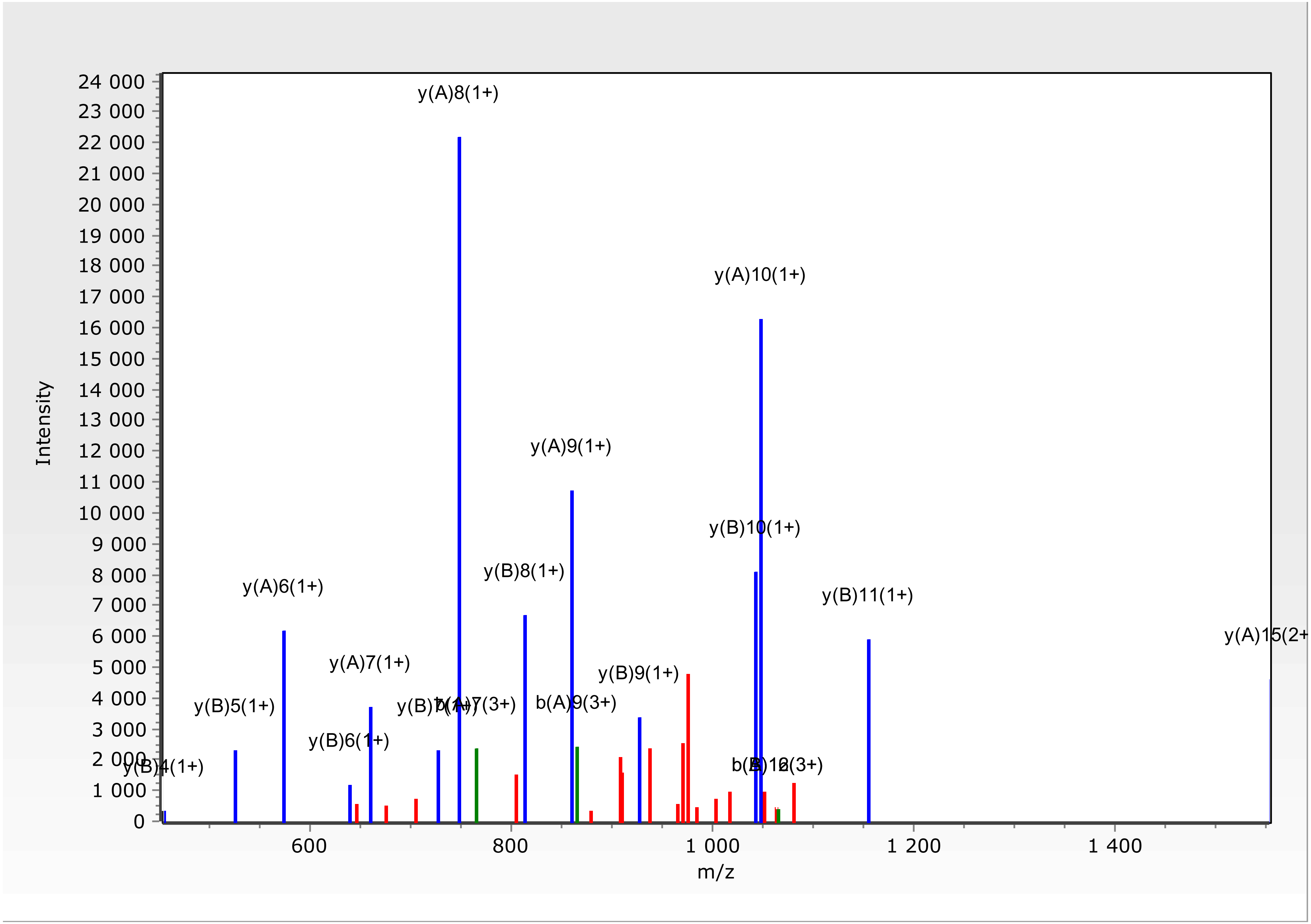

160218_ FXN[81-210] Dimer_BS2G, K152-K197, QTPNKQIWLSSPSSGPK + TKLDLSSLAYSGK

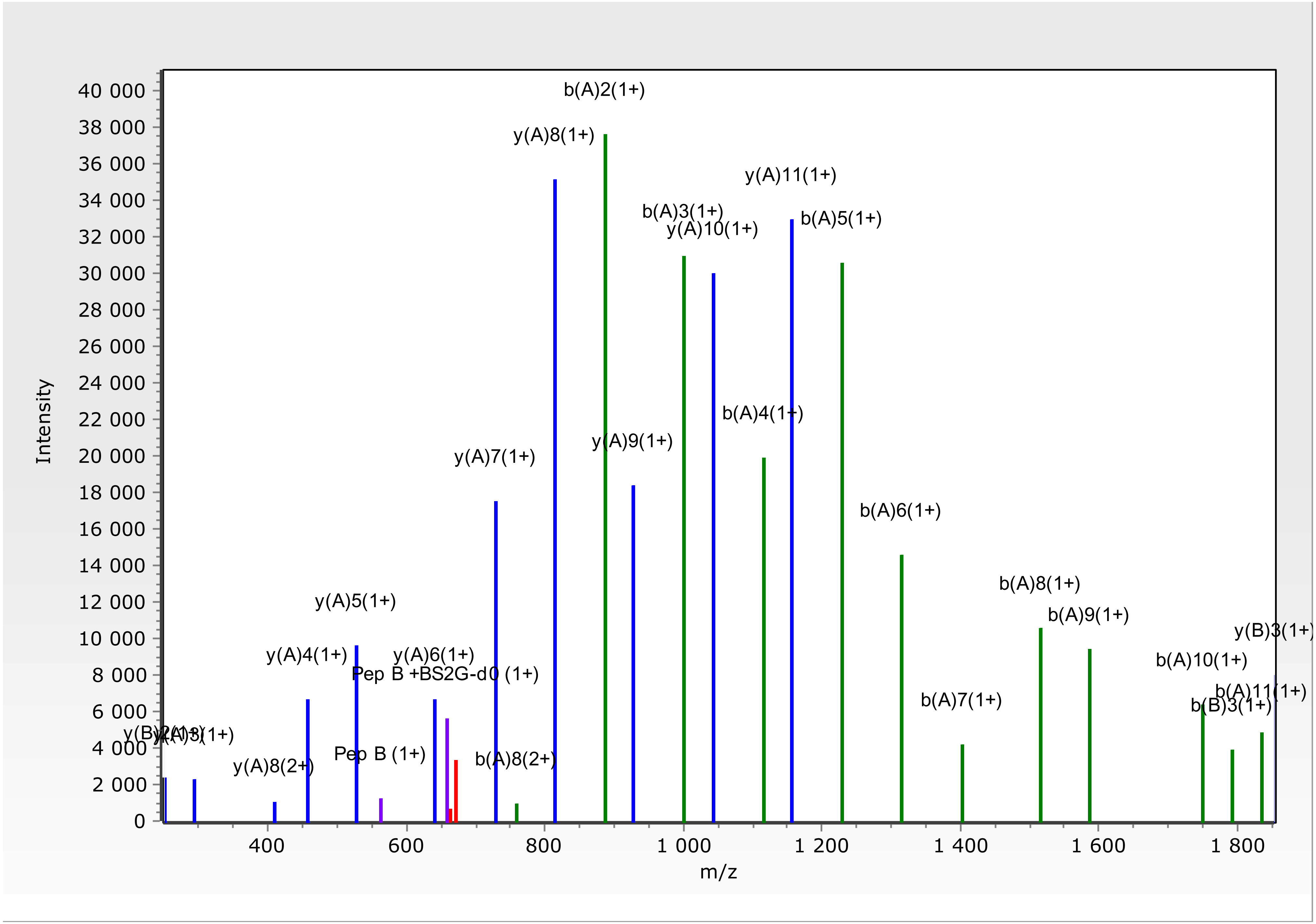

160218_ FXN$^{81\text{-}210}$ Dimer_BS2G, K197-K195, TKLDLSSLAYSGK + ALKTK

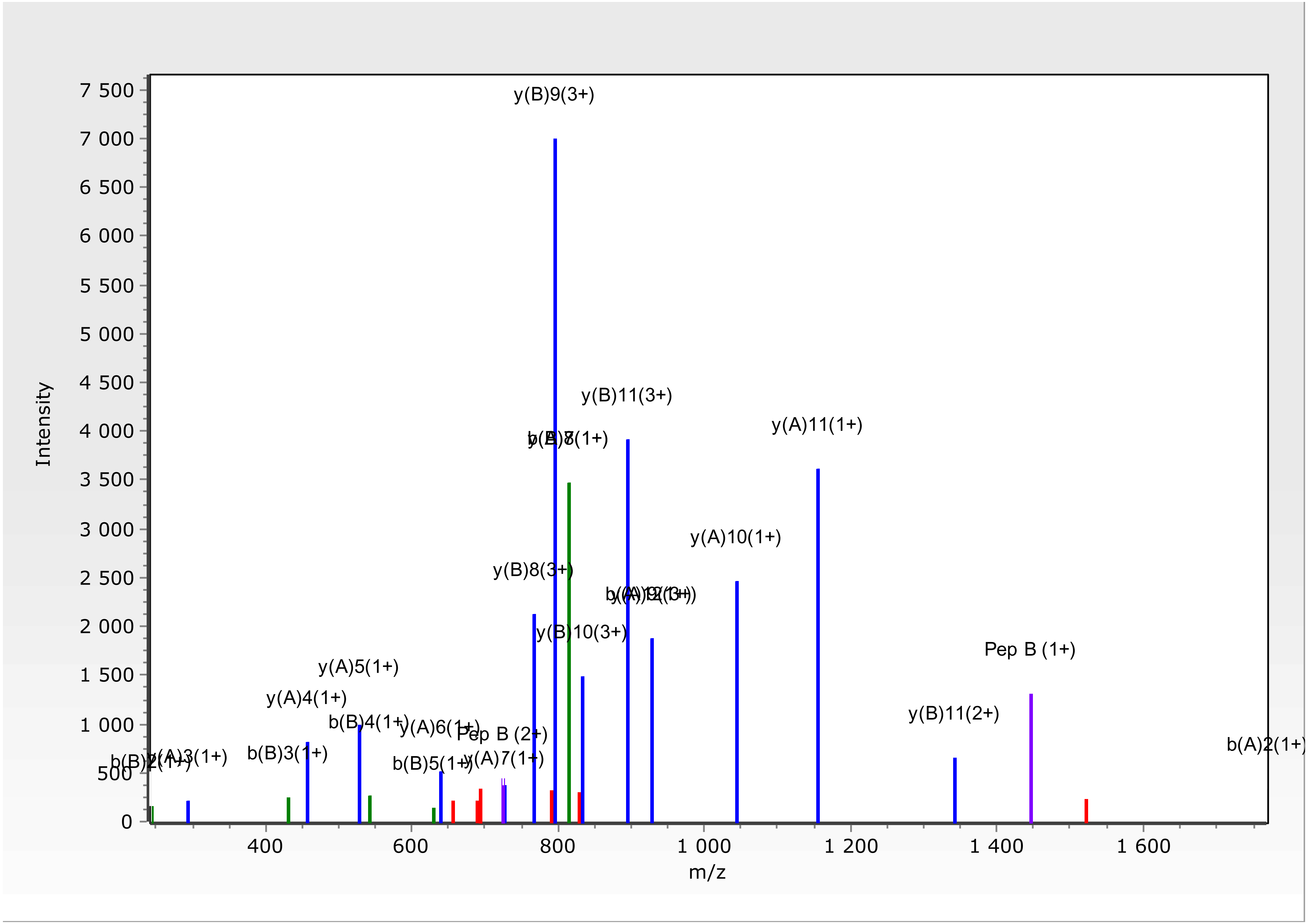

160218_ FXN$^{81-210}$ Dimer_BS2G, K197-K64, TKLDLSSLAYSGK + QIWLSSPSSGPKR

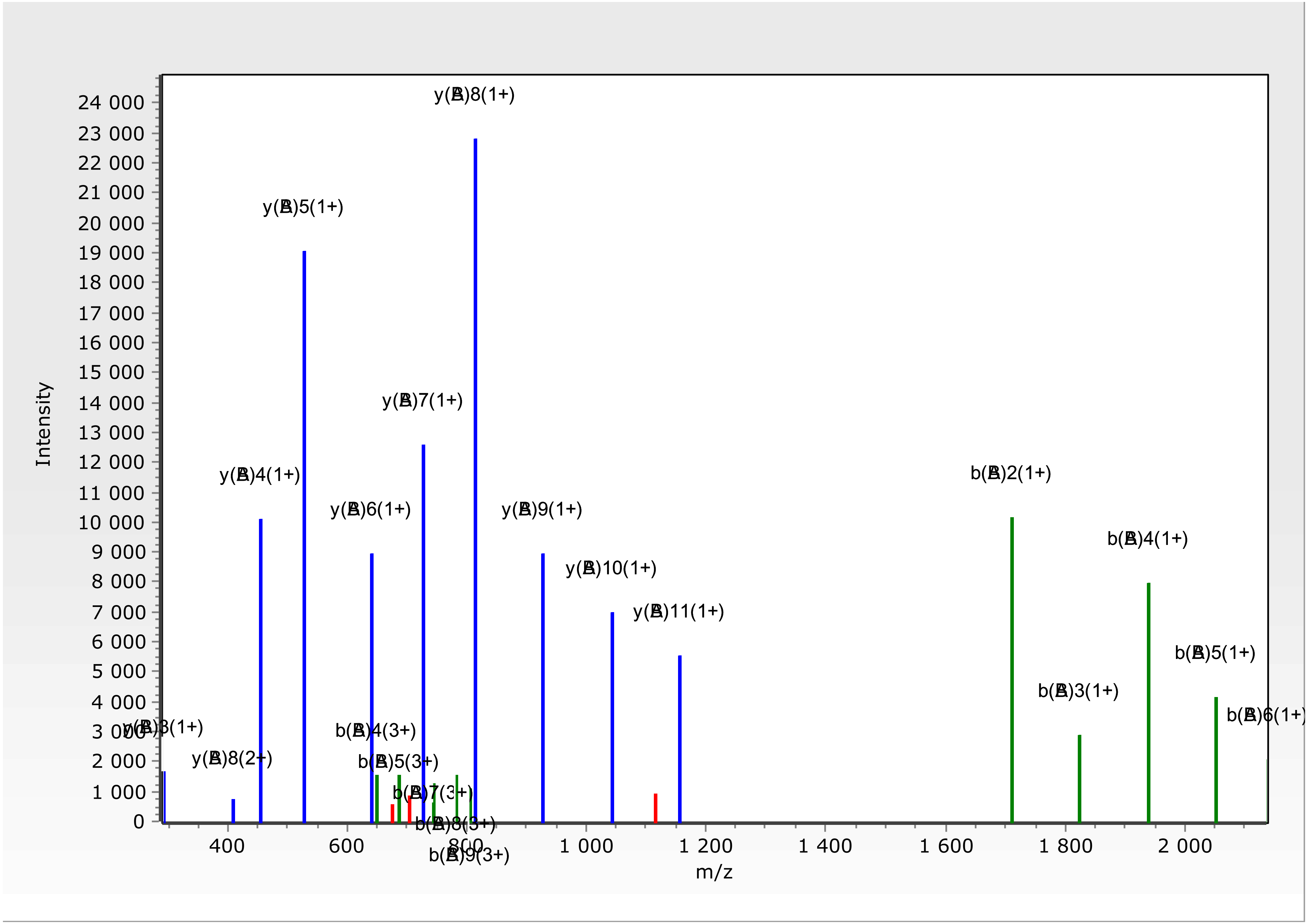

160218_ FXN$^{81\text{-}210}$ Dimer_BS2G, K197-K195, TKLDLSSLAYSGK + TKLDLSSLAYSGK

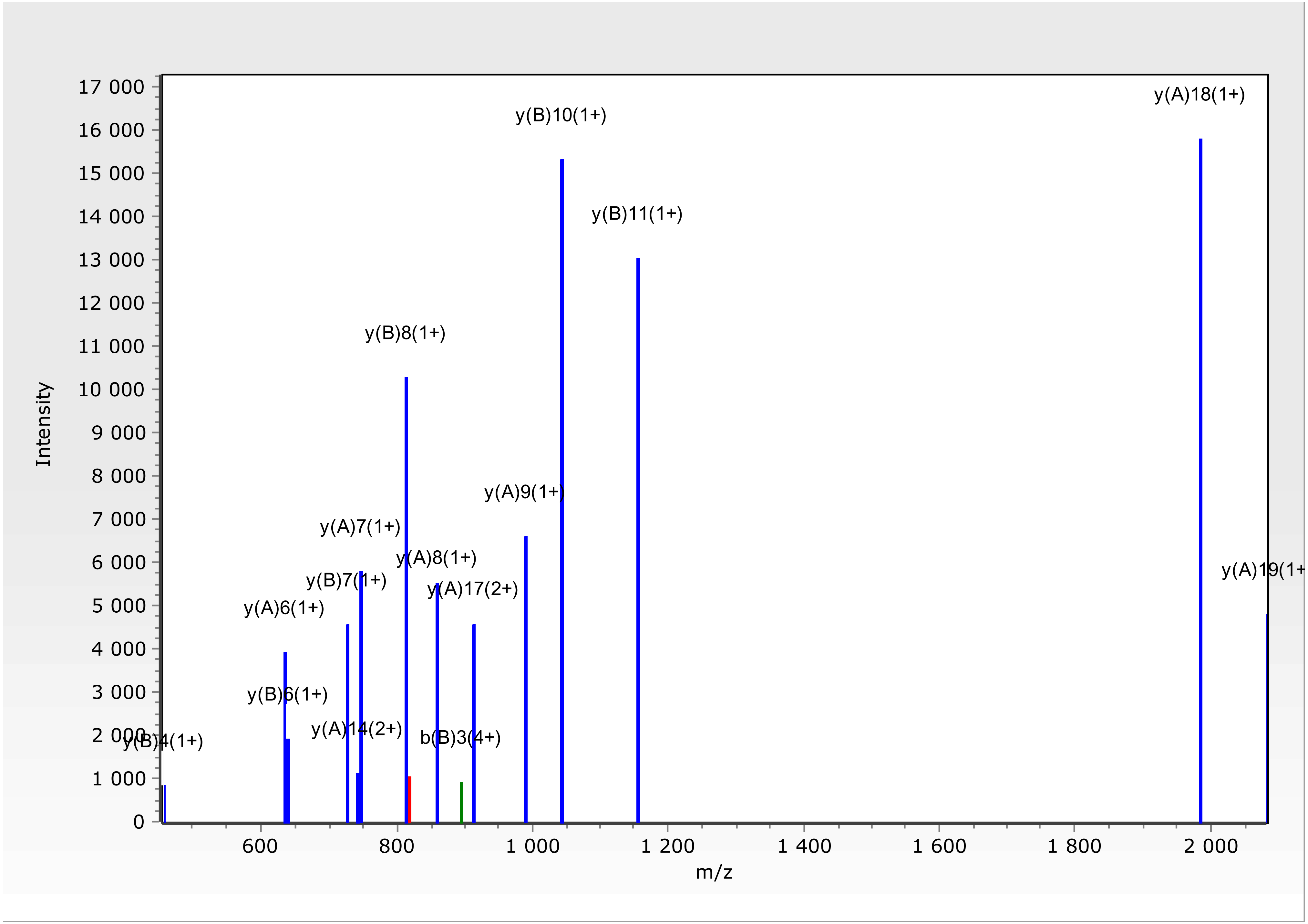

160218_FXN$^{81\text{-}210}$ Trimer_BS2G, K171-K197, YDWTGKNWVYSHDGVSLHELLAAELTK + TKLDLSSLAYSGK

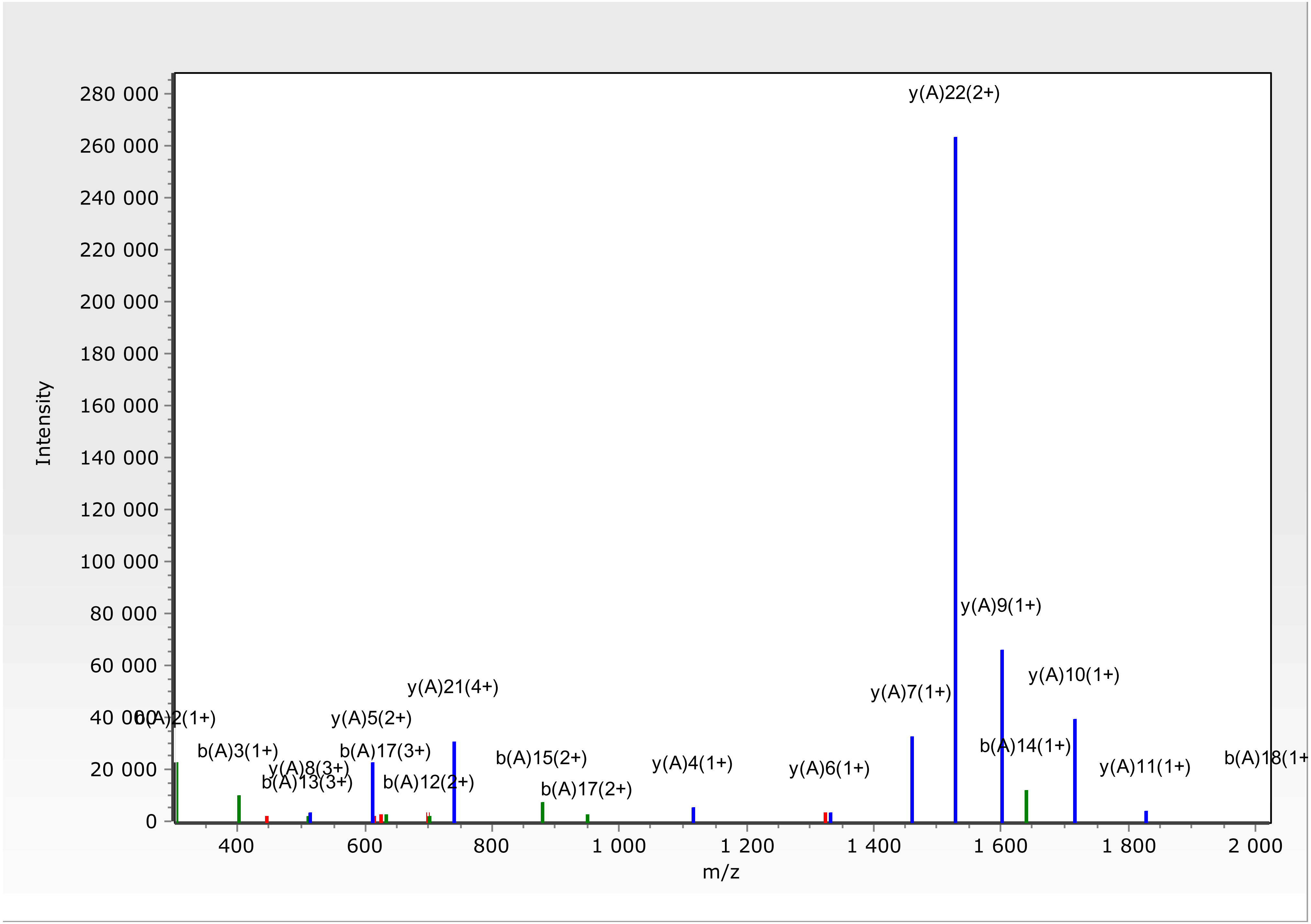

160218_ FXN$^{81\text{-}210}$ Trimer_BS2G, K192-K195, NWVYSHDGVSLHELLAAELTKALK + ALKTK

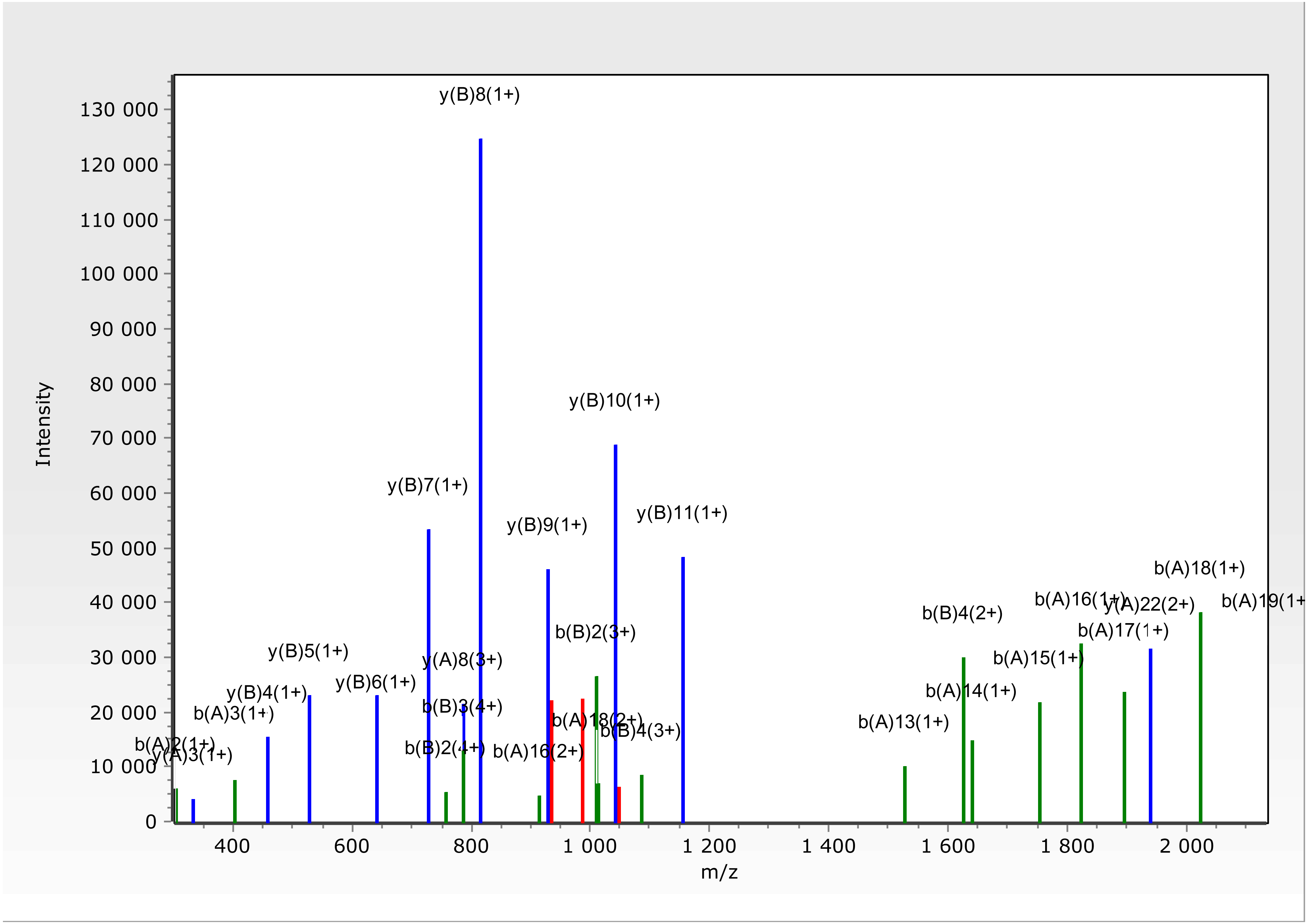

160218_ FXN$^{81\text{-}210}$ Trimer_BS2G, K192-K197, NWVYSHDGVSLHELLAAELTKALK + TKLDLSSLAYSGK

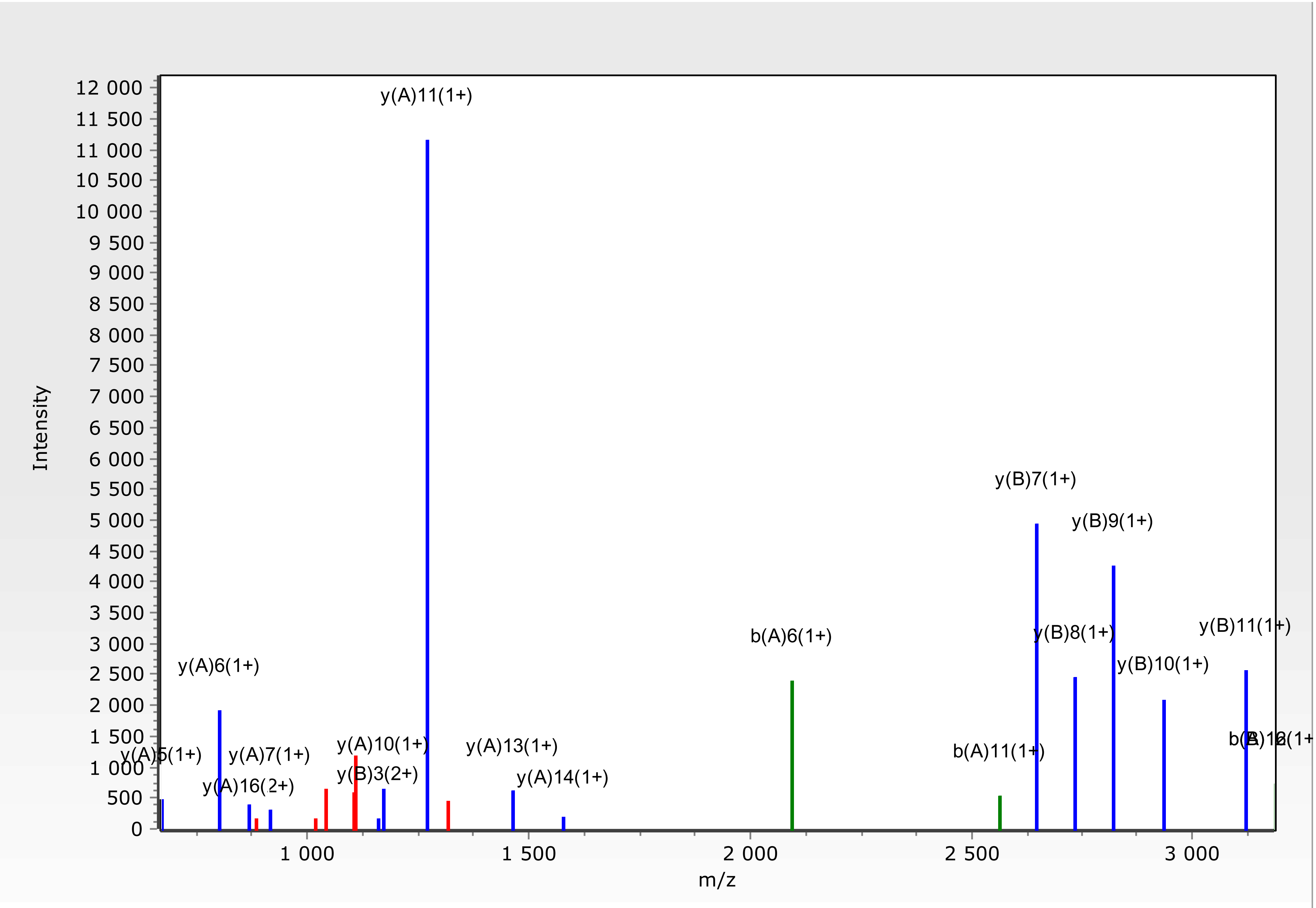

160218_FXN$^{81\text{-}210}$ Tetramer_BS2G, S81-K164, SGTLGHPGSLDETTYER + QIWLSSPSSGPKR

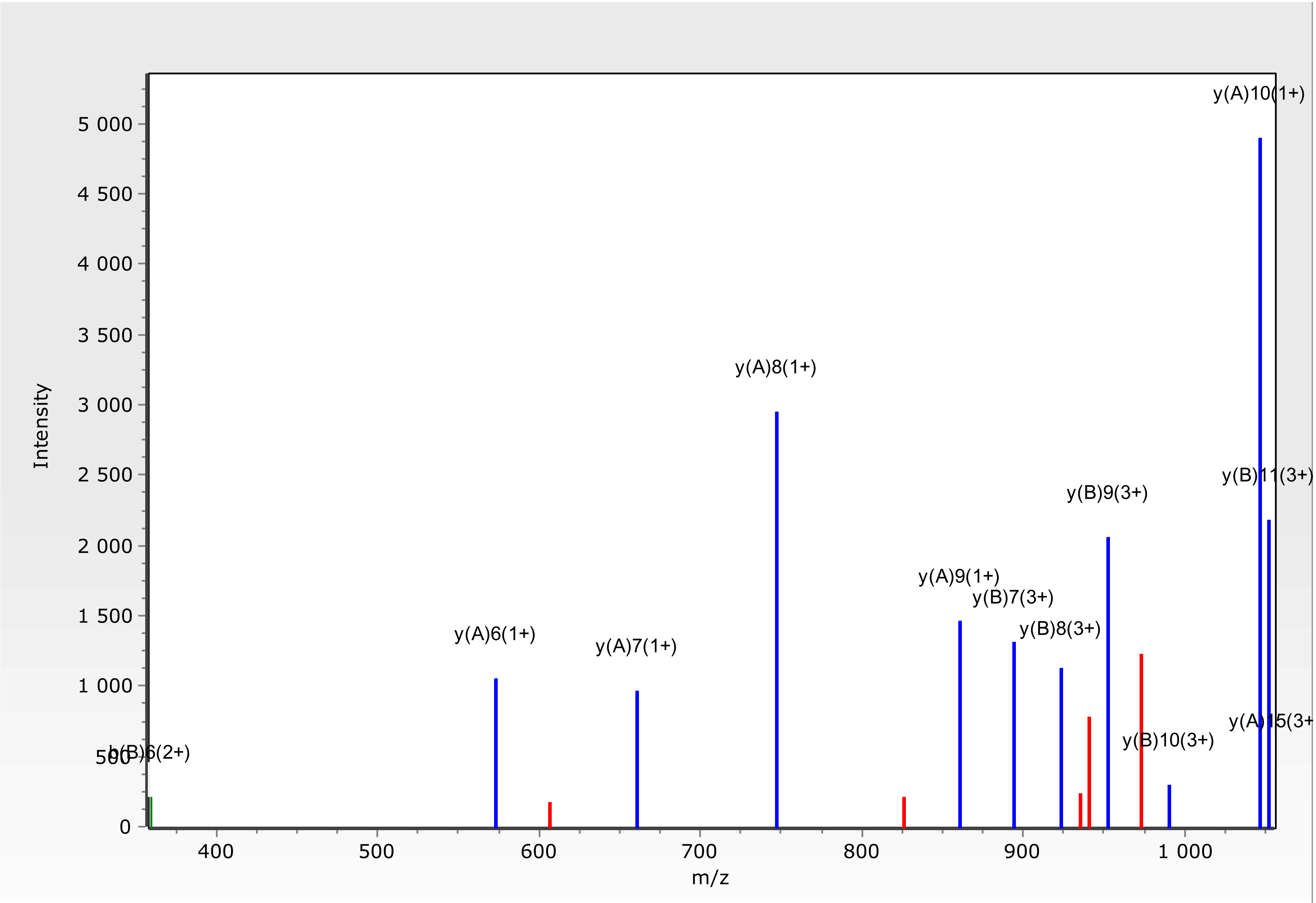

160218_ FXN^{81-210} Tetramer_BS2G, K152-K164, QTPNKQIWLSSPSSGPK + QIWLSSPSSGPKR

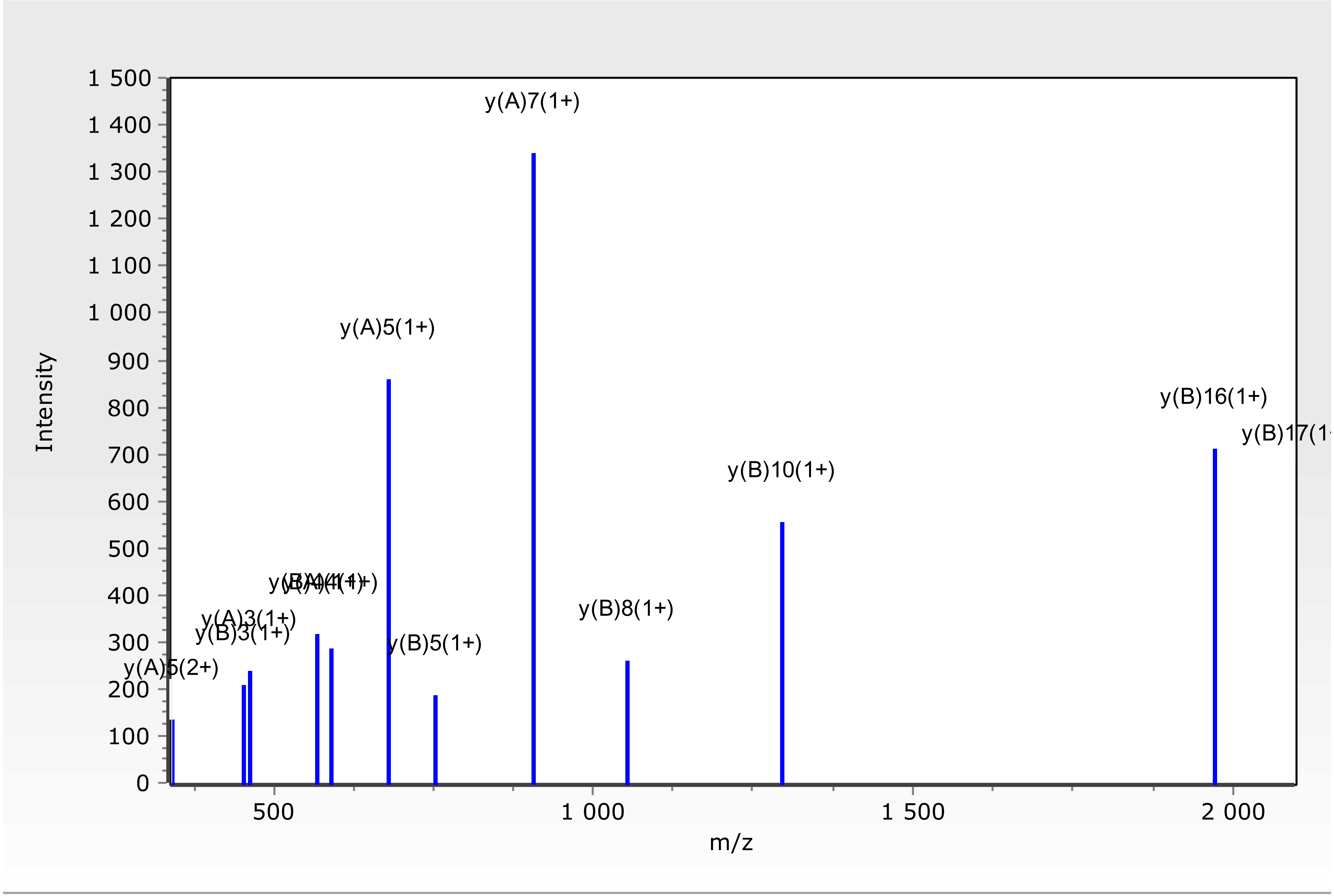

160221_CyaY_Dimer_BS2G, M1-K65 MNDSEFHR + QEPLHQVWLATKQGGYHFDLKGDEWICDR
